# Antioxidant and Anti-Inflammatory Constituents from the Roots of *Anodendron affine*: Inhibition of the fMLP-Induced Superoxide Anion Generation and Molecular Docking Studies

**DOI:** 10.3390/antiox15010097

**Published:** 2026-01-12

**Authors:** Shih-Jung Cheng, Yuen-Sing Lee, Lin-Yang Cheng, Sin-Min Li, Jih-Jung Chen

**Affiliations:** 1 School of Pharmacy, College of Pharmacy, Kaohsiung Medical University, Kaohsiung 807378, Taiwan; u105700005@kmu.edu.tw (S.-J.C.); andy.cheng@estrongmedical.com (L.-Y.C.); 2 Biomedical Industry Ph.D. Program, College of Life Sciences, National Yang Ming Chiao Tung University, Taipei 112304, Taiwan; ysl.ls11@nycu.edu.tw; 3 Department of Pharmacy, School of Pharmaceutical Sciences, National Yang Ming Chiao Tung University, Taipei 112304, Taiwan; sinminli@nycu.edu.tw; 4 Department of Medical Research, China Medical University Hospital, China Medical University, Taichung 404333, Taiwan; 5 Traditional Herbal Medicine Research Center, Taipei Medical University Hospital, Taipei 110301, Taiwan

**Keywords:** *Anodendron affine*, superoxide anion inhibition, oxidative stress, anti-inflammatory activity, molecular docking

## Abstract

Oxidative stress is a key driver of chronic inflammatory diseases. *Anodendron affine* is a native Formosan plant species in Taiwan that remains largely underexplored phytochemically and bioactivity. To reveal the bioactive constituents and assess its potential as a source of anti-inflammatory antioxidants, we performed bioactivity-guided fractionation and evaluated the inhibition of superoxide anion (O_2_^•−^) generation in formyl-L-methionyl-L-leucyl-L-phenylalanine-stimulated human neutrophils. Molecular docking simulations were employed to model interactions with Formyl peptide receptor 1 (FPR1) and the Nicotinamide adenine dinucleotide phosphate (NADPH) oxidase complex, including neutrophil cytosol factor 1 (p47phox) and NADPH oxidase 2 (NOX2), to propose a theoretical mechanism of action. Phytochemical investigation led to the isolation of two new compounds, methyl 4,5-*O*-diferuloyl-3-methoxyquinate (**1**) and 16-pregnen-3,12,20-trione (**2**), together with four known compounds. Notably, 4-hydroxy-3-prenylbenzoic acid (**5**) exhibited potent inhibitory activity (IC_50_ = 17.65 ± 0.97 μM), surpassing the activity of the positive control, ibuprofen (IC_50_ = 27.85 ± 3.56 μM). Docking studies suggested that anodendrosin H (**4**) and 4-hydroxy-3-prenylbenzoic acid (**5**) exhibit high predicted binding affinity to p47phox and NOX2. Based on these results, compounds **1**, **4**, and **5** from *A. affine* were identified as potential lead candidates for the development of novel anti-inflammatory therapeutics.

## 1. Introduction

*Anodendron affine* Druce (Apocynaceae) is an evergreen liana, distributed in Taiwan, the Ryukyu Islands, Japan, and southern China [1]. Taiwan possesses a rich and unique biodiversity, hosting a vast array of Formosan plants that have been utilized in traditional medicine for centuries. In our continued investigation of bioactive constituents from these native species [2], *A*. *affine* has attracted attention due to its diverse ethnobotanical applications [3]. Despite the traditional use of several Anodendron species [4,5], the specific anti-inflammatory constituents and molecular mechanisms of the native Formosan species *A. affine* remain largely unexplored. Therefore, this study aimed to investigate the chemical constituents from the roots of *A. affine* and to evaluate their potential as novel anti-inflammatory leads.

Inflammation is a protective response of cells to pathogen infection or tissue injury [6]. However, dysregulated inflammation becomes a critical driver of pathology with significant global health implications [7]. Chronic inflammation is increasingly recognized by the international medical community as a common underlying mechanism in widespread diseases, including cardiovascular pathologies, metabolic syndrome, autoimmune disorders, and cancer [8,9,10].

Oxidative stress, particularly the overproduction of reactive oxygen species (ROS) by neutrophils, plays a pivotal role in propagating this inflammatory damage [11]. While traditional non-steroidal anti-inflammatory drugs (NSAIDs) are widely used, the search for novel structural scaffolds from unique biodiversity remains a global priority to overcome efficacy limits and side effects [12,13]. Therefore, the discovery of specific inhibitors from under-explored indigenous species like *A. affine* that can target the neutrophil respiratory burst without compromising general immunity is of high relevance for developing new therapeutic strategies against these globally prevalent conditions.

Neutrophils, a type of phagocytic cell in the blood, are typically highly active during the acute phase of inflammation and are among the first responders to migrate to the site of inflammation [14]. Guided by chemical signals such as interleukin-8 (IL-8), C5a, leukotriene B4, and H_2_O_2_ through chemotaxis, neutrophils migrate through blood vessels and interstitial tissue [15]. Upon activation, neutrophils release reactive oxygen species (ROS), including the superoxide anion (O_2_^•−^) and hydroxyl radicals [16], as well as inflammatory mediators such as elastase, β-glucuronidase, lysozyme, and platelet-activating factor (PAF), which are associated with inflammatory diseases [17]. Therefore, suppressing the excessive or inappropriate activation of neutrophils through pharmacological agents is considered a potential strategy for improving inflammatory diseases.

Previous studies on natural products have shown that compounds, including nervonic acids [18,19,20], xanthones [21,22], flavonoids [23,24], coumarins [25,26,27], and lignans [28,29,30], possess anti-inflammatory activities. Our previous investigations have discovered numerous powerful bioactive compounds from natural sources [31]. Screening plants for anti-inflammatory activity enables the selection of promising species for further isolation and pharmacological evaluation, with the goal of discovering new leads for anti-inflammatory drugs.

**Figure 1:**
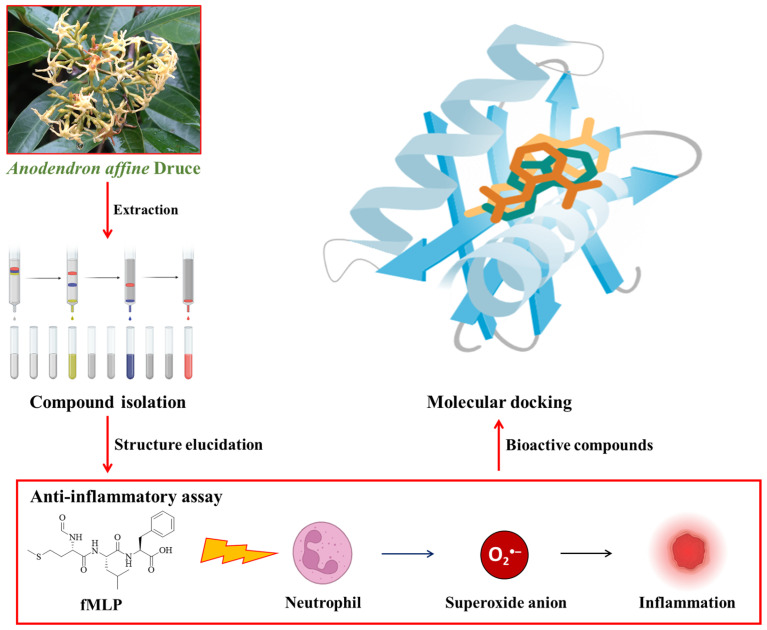
Conceptual framework of the study. The methanolic extract from the roots of *Anodendron affine* was subjected to bioactivity-guided isolation. The identified compounds were evaluated for their ability to inhibit superoxide anion generation in formyl-L-methionyl-L-leucyl-L-phenylalanine-stimulated human neutrophils. To predict the potential mechanism, molecular docking was performed against key targets of the neutrophil respiratory burst.

Since the methanolic extract from the roots of *A. affine* exhibited significant inhibitory activity (IC_50_ = 5.28 ± 0.43 μg/mL) against formyl-L-methionyl-L-leucyl-L-phenylalanine (fMLP)-induced superoxide (O_2_^•−^) generation in human neutrophils, we selected the roots of *A. affine* as our research material. This study aims to isolate its anti-inflammatory constituents, with the goal of contributing to the development of new anti-inflammatory agents. Figure 1 illustrates the overall conceptual framework of this study.

## 2. Materials and Methods

### 2.1. Chemicals and Reagents

All solvents used for extraction and isolation, including methanol (MeOH), ethyl acetate (EtOAc), *n*-hexane, acetone, *n*-butanol (*n*-BuOH), dichloromethane (CH_2_Cl_2_), and chloroform (CHCl_3_), were of analytical grade and purchased from Echo Chemical Co., Ltd. (Miaoli, Taiwan). Deuterated chloroform (CDCl_3_, 99.8%) containing 0.03% (*v*/*v*) tetramethylsilane (TMS) for Nuclear Magnetic Resonance (NMR) spectroscopy was obtained from Merck KGaA (Darmstadt, Hesse, Germany). Silica gel for column chromatography (Kieselgel 70–230 mesh and 230–400 mesh) and pre-coated silica gel 60 F254 plates for thin-layer chromatography (TLC) were also supplied by Merck KGaA (Darmstadt, Hesse, Germany). For the bioassay, the following chemicals were used: Hank’s Buffered Salt Solution (HBSS), formyl-L-methionyl-L-leucyl-L-phenylalanine-stimulated (fMLP), cytochalasin B (CB), ferricytochrome c, superoxide dismutase (SOD), phorbol 12-myristate 13-acetate (PMA), and ibuprofen. These were purchased from Sigma-Aldrich (St. Louis, MO, USA). All other chemicals and reagents were of the highest purity commercially available.

### 2.2. Plant Material

The roots of *A. affine* Druce were collected from Mudan Township, Pingtung County, Taiwan. A voucher specimen was authenticated and deposited at the Herbarium of the School of Pharmacy, China Medical University, Taichung, Taiwan.

### 2.3. General Experimental Procedures

Melting points were determined using a Yanaco MP-500D micro-melting point apparatus (Yanaco Technical Science Co., Ltd., Kyoto, Japan). Infrared (IR) spectra were recorded on a PerkinElmer System 2000 FT-IR spectrometer (PerkinElmer, Inc., Waltham, MA, USA). Ultraviolet (UV) spectra were measured on a Hitachi U-200 (Hitachi, Ltd., Tokyo, Japan) or a Jasco U-240 spectrophotometer (JASCO International Co., Ltd., Tokyo, Japan). Nuclear Magnetic Resonance (NMR) spectra, including ^1^H, ^13^C, DEPT, COSY, NOESY, HSQC, and HMBC experiments, were recorded on Varian Inova 500 (500 MHz for ^1^H) and Varian Unity Plus/Mercury 400 (400 MHz for ^1^H) spectrometers (Varian Medical Systems, Inc., Palo Alto, CA, USA), using tetramethylsilane (TMS) as an internal standard. Electrospray Ionization Mass Spectrometry (ESI-MS) and High-Resolution ESI-MS (HR-ESI-MS) data were obtained using a Bruker Autoflex III mass spectrometer (Bruker Daltonics, Billerica, MA, USA) and a VG Platform Electrospray instrument (Micromass UK Ltd., Manchester, UK). Column chromatography (CC) was performed using silica gel (Kieselgel 70–230 mesh and 230–400 mesh, Merck KGaA, Darmstadt, Hesse, Germany). Analytical and preparative thin-layer chromatography (TLC) was performed on pre-coated silica gel 60 F254 plates (0.2 mm and 0.5 mm thickness, respectively; Merck KGaA, Darmstadt, Hesse, Germany).

### 2.4. Extraction and Isolation

**Figure 2:**
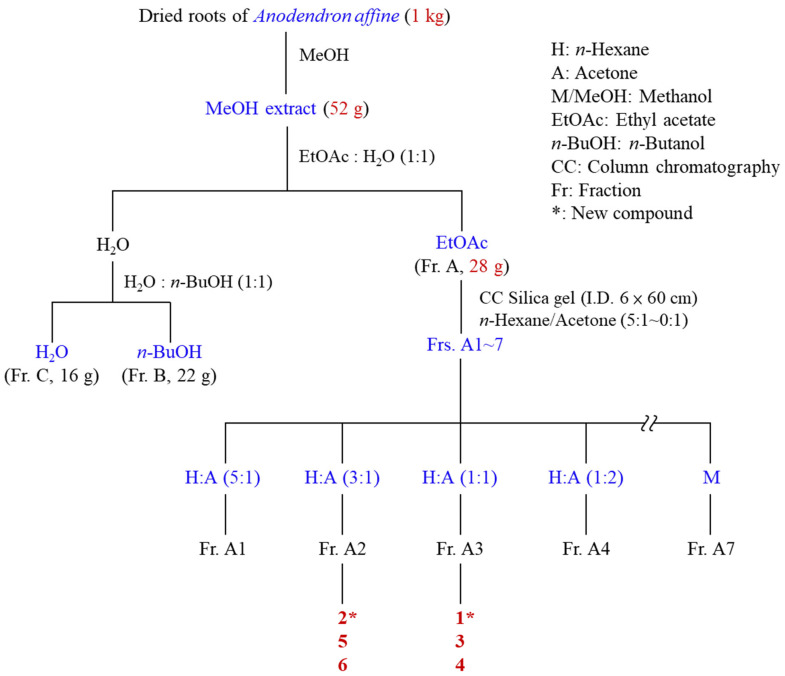
Flowchart of isolating compounds from *A. affine*.

The isolation procedure for *A. affine* compounds is outlined in Figure 2. The process began with the maceration of shade-dried, powdered roots (2.2 kg) in methanol (3 × 10 L, 7 days per extraction) at room temperature. The combined MeOH extracts were concentrated under reduced pressure to yield a crude residue (52 g). This residue was subsequently suspended in H_2_O and partitioned with ethyl acetate (EtOAc) and *n*-butanol (*n*-BuOH) to yield the EtOAc-soluble fraction (Fr. A, 28 g), the *n*-BuOH-soluble fraction (Fr. B, 22 g), and the aqueous fraction (Fr. C, 16 g). The EtOAc fraction (Fr. A, 28 g) was subjected to silica gel column chromatography and eluted with a stepwise gradient of *n*-hexane/acetone (from 5:1 to 0:1, *v*/*v*), followed by acetone and finally MeOH, to yield seven fractions (Fr. A1–A7). Fr. A2 (3.8 g), eluted with *n*-hexane/acetone (3:1), was further fractionated by silica gel CC (CH_2_Cl_2_/MeOH, 30:1→0:1) to give nine subfractions (Fr. A2-1 to A2-9). Fr. A2-3 (124 mg) was purified by MPLC (CHCl_3_/EtOAc, 6:1→0:1) and then by PTLC (CH_2_Cl_2_/EtOAc, 5:1) to yield compound **2** (3.2 mg). Fr. A2-5 (58 mg) was purified by PTLC (CH_2_Cl_2_/acetone, 5:1) to yield compound **6** (2.0 mg). Fr. A2-6 (38 mg) was purified by preparative TLC (CHCl_3_/MeOH, 15:1) to yield compound **5** (2.6 mg). Fr. A3 (5.5 g), eluted using *n*-hexane/acetone (1:1), was fractionated by silica gel CC (CH_2_Cl_2_/MeOH, 20:1→0:1) to give nine subfractions (Fr. A3-1 to A3-9). Fr. A3-4 (425 mg) was subjected to MPLC (CHCl_3_/MeOH, 20:1→0:1), and then Fr. A3-4-3 (46 mg) was purified by PTLC (CHCl_3_/EtOAc, 1:1) to yield compound **1** (2.8 mg). Fr. A3-7 (625 mg) was subjected to MPLC (CHCl_3_/MeOH, 15:1→0:1). Fr. A3-7-2 (30 mg) was purified by PTLC (CHCl_3_/acetone, 1:1) to yield compound **3** (2.2 mg). Fr. A3-7-4 (23 mg) was purified by PTLC (CHCl_3_/MeOH, 10:1) to yield compound **4** (2.8 mg).

### 2.5. Anti-Inflammatory Activity Assay (Superoxide Anion Generation in Human Neutrophils)

The anti-inflammatory activity of the isolated compounds was evaluated by measuring their inhibition of superoxide anion (O_2_^•−^) generation in fMLP-stimulated human neutrophils, as previously described [21] with minor modifications. Briefly, human neutrophils were isolated from the venous blood of healthy donors (20–28 years old) using a standard double-gradient Ficoll-Hypaque centrifugation method. Cell viability was confirmed to be greater than 95% by trypan blue exclusion. Neutrophils (6 × 10^5^ cells/mL) were pre-incubated with the test compounds (dissolved in DMSO, final concentration < 0.1%) or vehicle (DMSO) as a control at 37 °C for 5 min. Cells were then treated with cytochalasin B (CB, 5 µg/mL) for 3 min prior to stimulation with fMLP (1 µM). The superoxide anion production was quantified by measuring the superoxide dismutase-inhibitable reduction in ferricytochrome c at 550 nm using a Hitachi UV-3010 spectrophotometer (Hitachi, Ltd., Tokyo, Japan). The IC_50_ values were calculated from pooled donor data. Ibuprofen was used as a positive control.

### 2.6. Molecular Docking Study

Molecular docking simulations were performed to model the interaction between the compounds and enzymes according to established protocols [32]. The three-dimensional structures of the ligands were constructed with ChemDraw Ultra 20.0 (PerkinElmer, Waltham, MA, USA). The crystal structures of Formyl peptide receptor 1 (FPR1) in complex with an antagonist (PDB ID: 7T6T), the cytosolic subunit of Nicotinamide adenine dinucleotide phosphate (NADPH) oxidase, p47phox (1NG2), the catalytic subunit of NADPH oxidase, and NOX2 (gp91phox, PDB ID: 2CDU) were obtained from the Protein Data Bank. Hydrogen atoms were then incorporated into the protein structure. Both the protein and the compound structures were prepared for molecular docking. Gasteiger charge measurements and selection of flexible torsions for ligands were optimized by AutodockTools (ADT ver. 1.5.6) (Scripps Research, San Diego, CA, USA). Molecular docking studies were performed using AutoDock Vina software (ADT ver. 4.0.1) (Scripps Research, San Diego, CA, USA). The search grid was defined to encompass the active site of each receptor with the following parameters: FPR1 (7T6T) was centered at x = 106.9, y = 108.3, z = 120.9 with dimensions of 18 Å × 18 Å × 18 Å; p47phox (1NG2) was centered at x = 23.8, y = 41.6, z = 6.5 (18 Å × 18 Å × 18 Å); NOX2 (2CDU) was centered at x = 10.5, y = 4.1, z = 26.0 (40 Å × 40 Å × 40 Å). To ensure calculation accuracy, the exhaustiveness parameter was set to 20 with an energy range of 3 kcal/mol. The module calculated ten distinct docking conformations, which were ranked according to their predicted binding energy. The most stable docking conformations were visualized and analyzed using Discovery Studio 2019 (Dassault Systèmes, San Diego, CA, USA) to identify key amino acid residues involved in the predicted binding interactions.

### 2.7. Statistical Analysis

Data represent the mean ± SEM from a minimum of three independent experiments. Statistical significance, defined as *p* < 0.05, was determined by Student’s *t*-test. Data analysis was performed using SigmaPlot 14.0 (Systat Software Inc., San Jose, CA, USA).

## 3. Results and Discussion

### 3.1. Isolation and Structural Elucidation of Compounds

**Figure 3:**
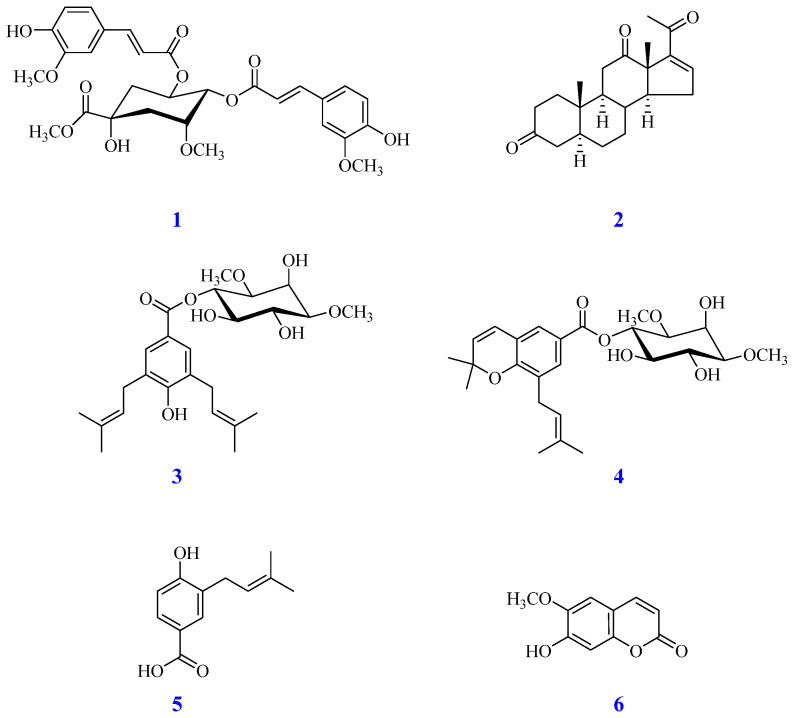
Compounds **1**–**6** isolated from the roots of *A. affine*.

Phytochemical investigation of the bioactive EtOAc-soluble fraction from roots of *A. affine* led to the isolation of six compounds **1**–**6**, as shown in Figure 3. The structures of the isolated compounds were determined using comprehensive spectroscopic analysis, including 1D/2D Nuclear Magnetic Resonance (NMR) techniques, and by comparing their data with existing literature. This process led to the identification of two new compounds, **1** and **2**, as well as four known compounds **3**–**6**.

### 3.2. Methyl 4,5-O-Diferuloyl-3-methoxyquinate (***1***)

**Figure 4:**
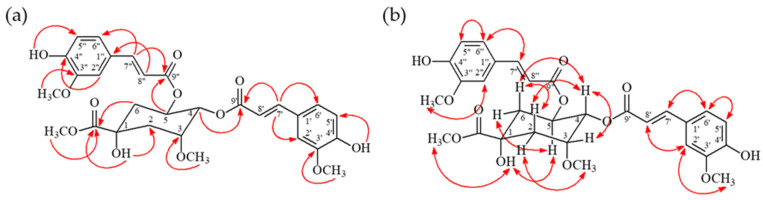
Key correlation relationship of HMBC (**a**) and NOESY (**b**) of compound **1**.

Compound **1** was obtained as an amorphous powder. Its molecular formula was established as C_31_H_36_O_12_ based on the HR-ESI-MS pseudo-molecular ion peak at *m*/*z* 623.21015 [M + Na]^+^ (calcd for C_31_H_36_O_12_Na, 623.21020). The IR spectrum showed absorptions for hydroxyl (3419 cm^−1^) and carbonyl (1704 cm^−1^) groups. The ^1^H- and ^13^C-NMR spectra (Figures S4 and S5) were highly similar to those of methyl 4-*O*-feruloyl-5-*O*-caffeoylquinate, with the key difference being the presence of two methoxy groups [δ_H_ 3.52 (3H, s) and 3.91 (3H, s); δ_C_ 59.7 and 56.0] in **1** instead of hydroxyl groups at the corresponding positions. This was confirmed by key HMBC correlations from the methoxy protons to C-3 (δ_C_ 78.6) and C-3′ (δ_C_ 146.7), respectively (Figure 4). The locations of the two feruloyl moieties at C-4 and C-5 of the quinic acid core were confirmed by HMBC correlations from H-4 (δ_H_ 5.16) and H-5 (δ_H_ 5.76) to the ester carbonyl carbons of the feruloyl groups (C-9″, δ_C_ 166.1 and C-9′, δ_C_ 166.7, respectively). The relative configuration of the quinic acid moiety was determined by analysis of NOESY correlations, which indicated axial orientations for H-4, H-5, and H-6, and equatorial orientations for H-2 and H-3. Based on this evidence, the structure of **1** was determined to be methyl 4,5-*O*-diferuloyl-3-methoxyquinate, which was confirmed to be a new compound via a SciFinder search.

### 3.3. 16-Pregnen-3,12,20-trione (***2***)

**Figure 5:**
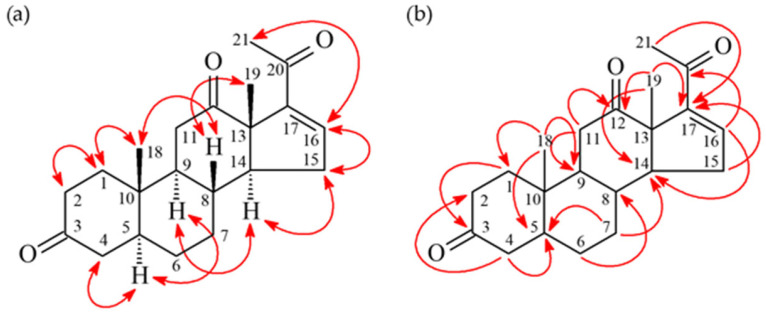
Key correlation relationship of NOESY (**a**) and HMBC (**b**) of compound **2**.

Compound **2** was isolated as an amorphous powder. HR-ESI-MS showed a pseudo-molecular ion at *m*/*z* 351.19291 [M + Na]^+^, consistent with the molecular formula C_21_H_28_O_3_ (calcd for C_21_H_28_O_3_Na, 351.19307). The IR spectrum indicated carbonyl (1711 cm^−1^) and conjugated carbonyl (1671 cm^−1^) groups. The ^1^H-NMR spectrum (Figures S13 and S14) displayed signals characteristic of a pregnane derivative, including two tertiary methyl singlets [δ_H_ 1.14 (3H, s, H-18), 1.36 (3H, s, H-19)], a methyl singlet adjacent to a carbonyl [δ_H_ 2.34 (3H, s, H-21)], and an olefinic proton [δ_H_ 6.62 (1H, dd, *J* = 3.2, 2.0 Hz, H-16)]. The NMR data were similar to those of 4,16-pregnadiene-3,12,20-trione [23], but differed by the absence of the Δ^4^ double bond. In **2**, H-4 and H-5 appeared as methane protons [δ_H_ 2.29 (m, H-4α), 2.16 (dd, *J* = 4.0, 2.0 Hz, H-4β), and 1.59 (m, H-5)], whereas they constituted a double bond in the known compound. This was confirmed by HMBC correlations (Figure 5) from H_2_-4 to C-2, C-3, C-5, C-6, and C-10, as well as a COSY correlation between H-4 and H-5. NOESY correlations indicated β-orientations for H-8 and the C-18/C-19 methyl groups, and α-orientations for H-5, H-9, and H-14. The structure of **2** was identified as a new compound by searching the SciFinder database, and named as 16-pregnen-3,12,20-trione.

### 3.4. Identification of Known Compounds

The four known compounds were identified as deglucosylanodendrosin A (**3**) [33], anodendrosin H (**4**) [33], 4-hydroxy-3-prenylbenzoic acid (**5**) [34], and Gelseminic acid (**6**) [35] by comparing their spectroscopic data (MS, NMR, IR) with those reported in the literature. Notably, compound **3** was isolated from a natural source for the first time, as it had previously been reported only as a hydrolysis product of anodendrosin A.

### 3.5. Anti-Inflammatory Activity

The isolated compounds (**1**–**6**) were evaluated for their anti-inflammatory potential by assessing their inhibition of formyl-L-methionyl-L-leucyl-L-phenylalanine-stimulated (fMLP)-induced superoxide anion (O_2_^•−^) in human neutrophils. Ibuprofen was selected as a positive control (IC_50_ = 27.85 ± 3.56 μM), as previously described [36]. The results are summarized in Table 1. Compounds **1**, **4**, and **5** exhibited significant inhibitory activity with IC_50_ values of 24.76 ± 0.45, 27.37 ± 2.28, and 17.65 ± 0.97 μM, respectively. The potency of compound **5** was comparable to that of the positive control ibuprofen. In contrast, compounds **2**, **3**, and **6** showed no significant activity (IC_50_ > 50 μM). The structure-activity relationship (SAR) among the nervogenic acid derivatives is noteworthy. Compound **4** (anodendrosin H), which possesses a 2,2-dimethyl-2H-chromene moiety, displayed more potent inhibition than its analogue **3** (deglucosylanodendrosin A), which lacks this structural feature. This suggests that the chromene ring may contribute to the anti-inflammatory activity. Furthermore, the prenylated benzoic acid derivative **5** was the most active compound identified in this study, indicating that this structural class holds promise for further investigation as anti-inflammatory leads. Cell viability remained above 90% among the compounds at the concentrations used for IC_50_ determination, suggesting that their inhibitory activities on superoxide production are unlikely to be primarily attributable to nonspecific cytotoxicity.

The present study confirms the potential of *A. affine* as a source of anti-inflammatory compounds. The isolated compounds represent diverse structural classes, including acylquinic acid derivatives (e.g., **1**), pregnanes (e.g., **2**), nervogenic acids (e.g., **3**, **4**), prenylbenzoic acids (e.g., **5**), and a coumarin (e.g., **6**), highlighting the chemical richness of this plant. The significant inhibitory effects of compounds **1**, **4**, and **5** on fMLP-induced superoxide anion production suggest that they may interfere with the activation pathway of neutrophils, potentially by inhibiting the assembly or activity of the NADPH oxidase complex. Further mechanistic studies are warranted to elucidate their precise molecular targets. The potent activity of compound **5** makes it a particularly attractive candidate for development as an anti-inflammatory lead agent.

### 3.6. Molecular Docking Analysis

To explore possible molecular interactions underlying the observed inhibition of fMLP-induced superoxide generation, molecular docking was performed as a supportive, hypothesis-generating approach. The selection of protein targets was based on their established and sequential roles in the fMLP-induced signaling pathway that leads to a burst of superoxide anion (O_2_^•−^), a primary reactive oxygen species (ROS) responsible for propagating oxidative stress and inflammation in human neutrophils.

Given that the biological assay measured the inhibition of fMLP-induced oxidative burst, the formyl peptide receptor 1 (FPR1), which is the direct and high-affinity target of the chemoattractant fMLP, was selected as the primary upstream target. FPR1 activation initiates the intracellular signaling cascade that triggers the massive generation of superoxide, a key driver of inflammatory tissue damage. The crystal structure of FPR1 in complex with an antagonist was obtained from the Protein Data Bank (PDB ID: 7T6T) [37].

Activation of FPR1 triggers downstream events that culminate in the assembly and activation of the NOX complex. A critical step is the phosphorylation and membrane translocation of the cytosolic organizer subunit p47phox. This protein acts as a central regulatory node, orchestrating the assembly of the active enzyme complex. Therefore, p47phox (PDB ID: 1NG2) was chosen as a key target to investigate potential inhibition of the ROS-producing enzyme assembly process. The catalytic core of the active complex is NOX2 (gp91phox), the membrane-bound subunit directly responsible for reducing oxygen to superoxide anions [38]. Docking against NOX2 (PDB ID: 2CDU) allowed for the evaluation of a direct inhibitory mechanism on the final effector of oxidative stress [39,40].

**Figure 6:**
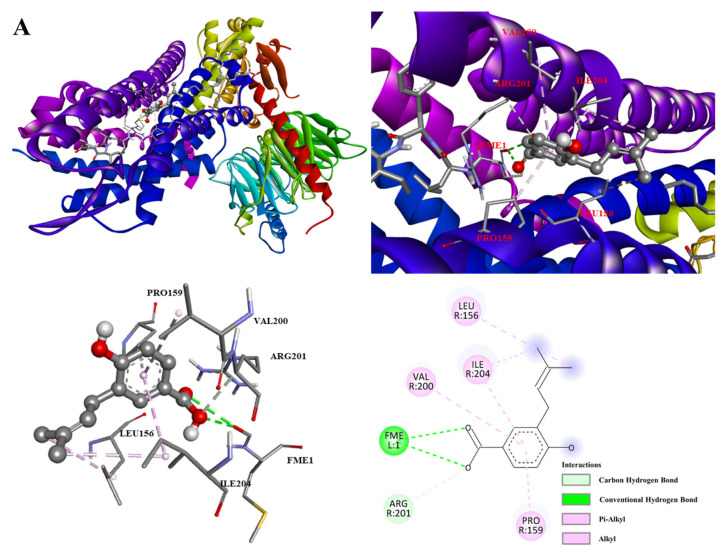

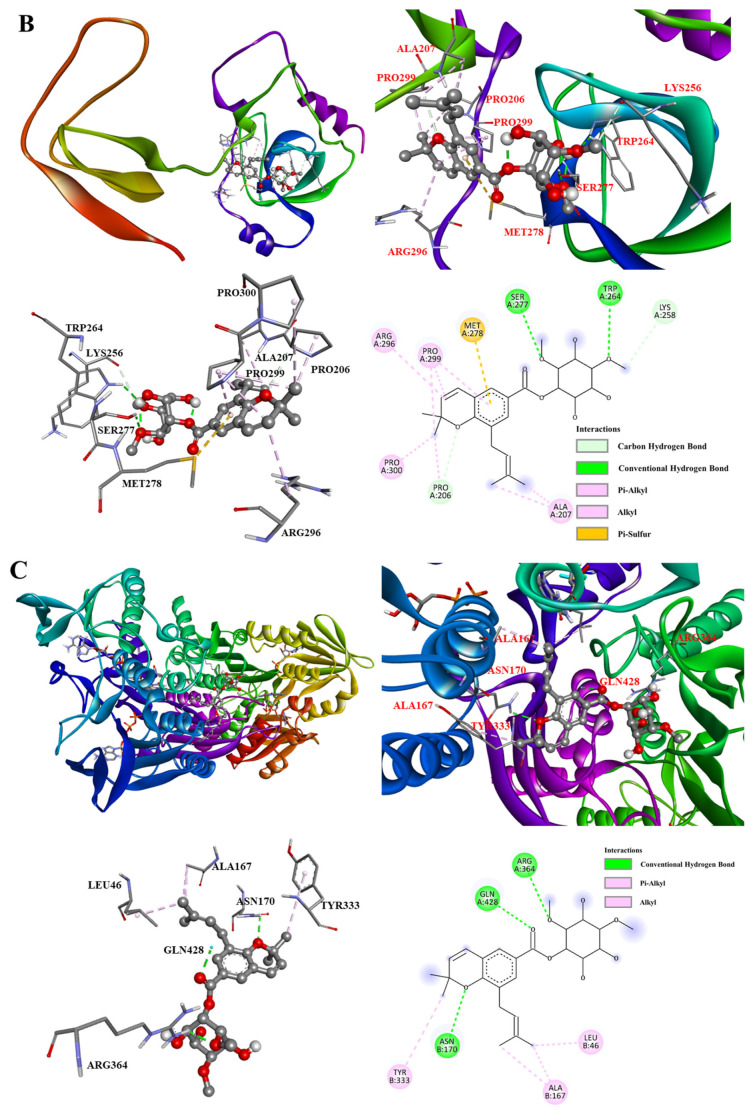
Molecular docking interactions. (**A**) Predicted binding mode of 4-hydroxy-3-prenylbenzoic acid (**5**) with formyl peptide receptor 1 (FPR1). (**B**) Predicted binding mode of anodendrosin H (**4**) with neutrophil cytosol factor 1 (p47phox). (**C**) Predicted binding mode of anodendrosin H (**4**) with NADPH oxidase 2 (NOX2). The purple halos around specific atoms in the 2D diagrams indicate solvent-accessible surfaces.

Compound **5** showed the strongest predicted binding affinity to FPR1 (−5.5 kcal/mol) over the reference compound ibuprofen (−4.9 kcal/mol), as detailed in Table 2. As illustrated in Figure 6A, the benzoic acid moiety of **5** formed alkyl and *π*–alkyl interactions with VAL200, ILE204, and PRO159. Additionally, conventional hydrogen bonds were observed between the carboxylic acid oxygen of the ligand and the residue with FME1. *π*–Alkyl interactions were also formed with ILE204 and LEU156 at the prenyl chain, stabilizing the complex.

In summary, the predicted high affinity of compound **5** for FPR1 can be attributed to this synergistic combination of hydrogen bonding and robust hydrophobic interactions, suggesting a potential mechanism for its antagonistic activity by occupying the receptor’s binding site.

The molecular docking results for anodendrosin H with the NADPH oxidase organizer subunit p47phox (PDB: 1NG2) are presented in Figure 6B. The predicted analysis revealed a high binding affinity of −7.9 kcal/mol, indicating a strong and stable interaction within the protein’s active site (Table 3).

The ligand forms key polar interactions, including a conventional hydrogen bond with the residue SER277 and TRP264. Additionally, carbon-hydrogen bonds are observed with the residues LYS258 and PRO206, which contribute significantly to the binding specificity and orientation. Hydrophobic interactions are a major stabilizing factor. The ligand engages in multiple alkyl and *π*–alkyl interactions with the residues ARG296, PRO300, ALA207, and PRO299, effectively anchoring its aliphatic regions within a hydrophobic pocket of the protein. A notable *π*–sulfur interaction is also present between an aromatic ring of the ligand and the sulfur atom of MET278, further reinforcing the complex’s stability.

The high binding affinity of anodendrosin H for p47phox is driven by a synergistic combination of directed hydrogen bonds and extensive hydrophobic contacts. This predicted binding mode suggests a potential mechanism by which the compound may inhibit the assembly and function of the NOX complex, thereby suppressing the production of superoxide anions.

The molecular docking results are shown in Table 4. Figure 6C shows the binding of anodendrosin H (**4**) to the NADPH oxidase catalytic subunit NOX2 (PDB: 2CDU). This compound exhibits the strongest binding affinity of −6.8 kcal/mol. Analysis prediction of the binding pose reveals that anodendrosin H engages with multiple subunits of the NOX2 complex through a combination of specific hydrogen bonding and hydrophobic interactions.

The binding is stabilized by several key conventional hydrogen bonds. The ligand forms these polar interactions with residues ARG364 and GLN428 from one subunit, and with ASN170 from an adjacent subunit, indicating the ligand’s ability to bridge different regions of the protein complex. Furthermore, the complex is stabilized by extensive hydrophobic interactions. The ligand participates in alkyl and *π*–alkyl interactions with multiple hydrophobic residues, including LEU46, ALA167, and TYR333. These interactions suggest that the non-polar regions of anodendrosin H are effectively accommodated within hydrophobic pockets of the enzyme.

The high binding affinity of compound **4** for NOX2 is predicted to be driven by a synergistic combination of multiple, specific hydrogen bonds that provide directionality, complemented by a series of hydrophobic interactions that enhance the overall binding stability. This binding mode positions the compound as a potential inhibitor of the enzyme’s catalytic activity, thereby supporting the experimental findings related to the suppression of superoxide anion generation. The molecular docking results provide a theoretical structural rationale for the experimentally observed inhibition of fMLP-induced O_2_^•−^ generation by compounds from *A. affine*. The analysis reveals distinct yet complementary mechanisms of action against key targets in the NADPH oxidase pathway.

### 3.7. Strengths and Limitations of the Study

The main strengths of this study are the isolation and identification of two novel compounds (**1** and **2**) from *A. affine* and the discovery that 4-hydroxy-3-prenylbenzoic acid (**5**) exhibits superior anti-inflammatory activity compared to the standard drug ibuprofen. Moreover, the use of primary human neutrophils ensures that these findings are physiologically relevant, confirming that the active compounds are membrane-permeable and effective in a functional cellular environment. Furthermore, this study combines functional cell experiments with multi-target molecular docking, providing a theoretical framework for the inhibition of oxidative stress. However, this study also has some limitations. Currently, the molecular mechanisms involved in the interactions of FPR1, NOX2, and p47phox are based on computer simulations. While molecular docking provides a theoretical structural explanation for the observed activities, direct biochemical enzyme inhibition experiments have not yet been performed. Future research will focus on using purified enzyme systems to validate these specific protein–ligand interactions and confirm their binding kinetics.

## 4. Conclusions

In conclusion, the phytochemical investigation from the roots of *Anodendron affine* led to the isolation of two new compounds, methyl 4,5-*O*-diferuloyl-3-methoxyquinate (**1**) and 16-pregnen-3,12,20-trione (**2**), along with four known compounds. The bioassay demonstrated significant anti-inflammatory activity for three compounds, including 4-hydroxy-3-prenylbenzoic acid (**5**) (IC_50_ = 17.65 ± 0.97 μM), methyl 4,5-*O*-diferuloyl-3-methoxyquinate (**1**) (IC_50_ = 24.76 ± 0.45 μM), and anodendrosin H (**4**) (IC_50_ = 27.37 ± 2.28 μM), all of which were comparable to or more potent than the reference compound ibuprofen (IC_50_ = 27.85 ± 3.56 μM). Molecular docking simulations predicted binding energies for compound **4** with p47phox (−7.9 kcal/mol) and NOX2 (−6.8 kcal/mol), pointing to a theoretical interaction. This suggests that the bioactive constituents may disrupt NADPH oxidase function by potentially targeting these key regulatory proteins. These findings suggest that bioactive compounds from *A. affine*, particularly compounds **1**, **4**, and **5**, warrant further investigation as promising lead candidates for the development of new anti-inflammatory agents. Future studies will focus on direct biochemical enzyme assays to validate the specific molecular targets and detailed kinetic analyses to elucidate their precise mechanism of action.

## Figures and Tables

**Table 1:** Inhibitory effects of **1**–**6** from the roots of *A. affine* on superoxide anion generation by human neutrophils in response to fMet-Leu-Phe.

Compounds	IC_50_ (μM) ^a^
Methyl 4,5-*O*-feruloyl-3-methoxyquinate (**1**)	24.76 ± 0.45 *
16-Pregnen-3,12,20-trione (**2**)	>50
Deglucosylanodendrosin A (**3**)	>50
Anodendrosin H (**4**)	27.37 ± 2.28 *
4-Hydroxy-3-prenylbenzoic acid (**5**)	17.65 ± 0.97 ***
Gelseminic acid (**6**)	>50
Ibuprofen ^b^	27.85 ± 3.56

^a^ The IC_50_ values were derived from the dose–response curves using SigmaPlot 14.0. Results represent the mean ± SEM (n = 3). ^b^ Ibuprofen was used as a positive control. * *p* < 0.01, *** *p* < 0.001 compared with the control.

**Table 2:** Predicted binding energies of bioactive compounds and ibuprofen against formyl peptide receptor 1 (FPR1).

Compounds	Affinity (kcal/mol)
Methyl 4,5-*O*-feruloyl-3-methoxyquinate (**1**)	−4.4
Anodendrosin H (**4**)	−4.1
4-Hydroxy-3-prenylbenzoic acid (**5**)	−5.5
Ibuprofen ^a^	−4.9

^a^ Ibuprofen was used as a positive control.

**Table 3:** Predicted binding energies of bioactive compounds and ibuprofen against neutrophil cytosol factor 1 (p47phox).

Compounds	Affinity (kcal/mol)
Methyl 4,5-*O*-feruloyl-3-methoxyquinate (**1**)	−6.3
Anodendrosin H (**4**)	−7.9
4-Hydroxy-3-prenylbenzoic acid (**5**)	−5.8
Ibuprofen ^a^	−5.5

^a^ Ibuprofen was used as a positive control.

**Table 4:** Predicted binding energies of bioactive compounds and ibuprofen against NADPH oxidase 2 (NOX2).

Compounds	Affinity (kcal/mol)
Methyl 4,5-*O*-feruloyl-3-methoxyquinate (**1**)	−6.5
Anodendrosin H (**4**)	−6.8
4-Hydroxy-3-prenylbenzoic acid (**5**)	−6.4
Ibuprofen ^a^	−5.5

^a^ Ibuprofen was used as a positive control.

## Data Availability

The original contributions presented in this study are included in the article/Supplementary Material. Further inquiries can be directed to the corresponding author.

## Supplementary Materials

The following supporting information can be downloaded at: https://www.mdpi.com/article/10.3390/antiox15010097/s1, Spectrum data of isolated compounds: Figures S1–S9: spectroscopic data of compound **1**. Figures S10–S19: spectroscopic data of compound **2**. Figures S20–S29: spectroscopic data of compound **3**. Figures S30–S32: spectroscopic data of compound **4**. Figures S33–S35: spectroscopic data of compound **5**. Figures S36–S38: spectroscopic data of compound **6**. Figure S1. ESI-MS spectrum of Methyl 4,5-*O*-feruloyl-3-methoxyquinate (**1**). Figure S2. HR-ESI-MS spectrum of Methyl 4,5-*O*-feruloyl-3-methoxyquinate (**1**). Figure S3. IR spectrum of Methyl 4,5-*O*-feruloyl-3-methoxyquinate (**1**). Figure S4. ^1^H-NMR spectrum of Methyl 4,5-*O*-feruloyl-3-methoxyquinate (**1**). Figure S5. ^13^C-NMR spectrum of Methyl 4,5-*O*-feruloyl-3-methoxyquinate (**1**). Figure S6. NOESY spectrum of Methyl 4,5-*O*-feruloyl-3-methoxyquinate (**1**). Figure S7. HMBC spectrum of Methyl 4,5-*O*-feruloyl-3-methoxyquinate (**1**). Figure S8. HSQC spectrum of Methyl 4,5-*O*-feruloyl-3-methoxyquinate (**1**). Figure S9. ^1^H-^1^H COSY spectrum of Methyl 4,5-*O*-feruloyl-3-methoxyquinate (**1**). Figure S10. ESI-MS spectrum of 16-Pregnen-3,12,20-trione (**2**). Figure S11. HR-ESI-MS spectrum of 16-Pregnen-3,12,20-trione (**2**). Figure S12. IR spectrum of 16-Pregnen-3,12,20-trione (**2**). Figure S13. ^1^H-NMR spectrum of 16-Pregnen-3,12,20-trione (**2**). Figure S14. ^13^C-NMR spectrum of 16-Pregnen-3,12,20-trione (**2**). Figure S15. NOESY spectrum of 16-Pregnen-3,12,20-trione (**2**). Figure S16. HMBC spectrum of 16-Pregnen-3,12,20-trione (**2**). Figure S17. HSQC spectrum of 16-Pregnen-3,12,20-trione (**2**). Figure S18. DEPT spectrum of 16-Pregnen-3,12,20-trione (**2**). Figure S19. ^1^H-^1^H COSY spectrum of 16-Pregnen-3,12,20-trione (**2**). Figure S20. ESI-MS spectrum of Deglucosylanodendrosin A (**3**). Figure S21. HR-ESI-MS spectrum of Deglucosylanodendrosin A (**3**). Figure S22. IR spectrum of Deglucosylanodendrosin A (**3**). Figure S23. ^1^H-NMR spectrum of Deglucosylanodendrosin A (**3**). Figure S24. ^13^C-NMR spectrum of Deglucosylanodendrosin A (**3**). Figure S25. NOESY spectrum of Deglucosylanodendrosin A (**3**). Figure S26. HMBC spectrum of Deglucosylanodendrosin A (**3**). Figure S27. HSQC spectrum of Deglucosylanodendrosin A (**3**). Figure S28. DEPT spectrum of Deglucosylanodendrosin A (**3**). Figure S29. ^1^H-^1^H COSY spectrum of Deglucosylanodendrosin A (**3**). Figure S30. ESI-MS spectrum of Anodendrosin H (**4**). Figure S31. IR spectrum of Anodendrosin H (**4**). Figure S32. ^1^H-NMR spectrum of Anodendrosin H (**4**). Figure S33. ESI-MS spectrum of 4-Hydroxy-3-prenylbenzoic acid (**5**). Figure S34. IR spectrum of 4-Hydroxy-3-prenylbenzoic acid (**5**). Figure S35. ^1^H-NMR spectrum of 4-Hydroxy-3-prenylbenzoic acid (**5**). Figure S36. ESI-MS spectrum of Gelseminic acid (**6**). Figure S37. IR spectrum of Gelseminic acid (**6**). Figure S38. ^1^H-NMR spectrum of Gelseminic acid (**6**).
