# Supplementary material for: Antioxidant and Anti-Inflammatory Constituents from the Roots of Anodendron affine: Inhibition of the fMLP-Induced Superoxide Anion Generation and Molecular Docking Studies
Source: Antioxidants (Basel). 2026 Jan 12;15(1):97. doi: 10.3390/antiox15010097 (PMC12837968; doi:10.3390/antiox15010097)
Supplement: Supplementary file 1 [file antioxidants-15-00097-s001.zip › Supplementary Information.pdf]

Supporting Information

Antioxidant and Anti-Inflammatory Constituents from the Roots of *Anodendron affine*: Inhibition of the fMLP-Induced Superoxide Anion Generation and Molecular Docking Studies

Shih-Jung Cheng [1,†], Yuen-Sing Lee [2,†], Lin-Yang Cheng [3], Sin-Min Li [4], and Jih-Jung Chen [2,4,5,6,*]

[1] School of Pharmacy, College of Pharmacy, Kaohsiung Medical University, Kaohsiung 807378, Taiwan; lovecola218@gmail.com (S.-J.C.)
[2] Biomedical Industry Ph.D. Program, College of Life Sciences, National Yang Ming Chiao Tung University, Taipei 112304, Taiwan; ysl.ls11@nycu.edu.tw (Y.-S.L.)
[3] School of Pharmacy, College of Pharmacy, Kaohsiung Medical University, Kaohsiung 807378, Taiwan; andy.cheng@estrongmedical.com (L.-Y.C.)
[4] Department of Pharmacy, School of Pharmaceutical Sciences, National Yang Ming Chiao Tung University, Taipei 112304, Taiwan; samuel147samuel147@gmail.com (S.-M.L.)
[5] Department of Medical Research, China Medical University Hospital, China Medical University, Taichung 404333, Taiwan
[6] Traditional Herbal Medicine Research Center, Taipei Medical University Hospital, Taipei 110301, Taiwan
* Correspondence: jjungchen@nycu.edu.tw; Tel.: +886-2-2826-7195; Fax: +886-2-2823-2940;
† These authors contributed equally to this work.

Contents

Spectrum data of isolated compounds……S3

MS, IR, and NMR spectrum of isolated compound **1**……S6

MS, IR, and NMR spectrum of isolated compound **2**……S10

MS, IR, and NMR spectrum of isolated compound **3**……S15

MS, IR, and NMR spectrum of isolated compound **4**……S20

MS, IR, and NMR spectrum of isolated compound **5**……S22

MS, IR, and NMR spectrum of isolated compound **6**……S23

Spectrum data of isolated compounds

Methyl 4,5-*O*-feruloyl-3-methoxyquinate (**1**): amorphous powder; UV (MeOH) λ_{max} (log ε) 218 (4.09), 237 (4.01), 329 (4.28) nm; IR (KBr) υ_{max} 3419 (OH), 1704 (C=O) cm^{-1}; ^{1}H-NMR ($CDCl_3$, 500 MHz) δ 2.10 (1H, m, H_{ax}-6), 2.15 (1H, m, H_{ax}-2), 2.34 (1H, dt, *J* = 14.5, 3.0 Hz, H_{eq}-2), 2.51 (1H, dt, *J* = 13.0, 5.0 Hz, H_{eq}-6), 3.52 (3H, s, OMe-3), 3.79 (3H, s, OMe-7), 3.89 (3H, s, H-3′), 3.91 (3H, s, H-3″), 4.08 (1H, d, *J* = 3.0 Hz, H_{eq}-3), 4.47 (1H, br s, OH-1, D_2O exchangable), 5.16 (1H, dd, *J* = 10.0, 3.0 Hz, H_{ax}-4), 5.76 (1H, td, *J* = 10.0, 5.0 Hz, H_{ax}-5), 5.83 (1H, br s, OH-4′, D_2O exchangable), 5.86 (1H, br s, OH-4″, D_2O exchangable), 6.21 and 6.29 (each 1H, d, *J* = 16.0 Hz, H-8″ and 8′), 6.88 and 6.89 (each 1H, d, *J* = 8.0 Hz, H-5′ and 5″), 6.97 (1H, d, *J* = 1.0 Hz, H-2″), 7.01 (1H, br s, H-2′), 7.04 (2H, dd, *J* = 8.0, 1.0 Hz, H-6′ and 6″), 7.57 and 7.64 (each 1H, d, *J* = 16.0 Hz, H-7″ and 7′); ^{13}C-NMR ($CDCl_3$, 125 MHz) δ 35.1 (C-2), 39.7 (C-6), 52.9 (OMe-7), 55.9 (OMe-3′), 56.0 (OMe-3″), 59.7 (OMe-3), 66.9 (C-5), 74.6 (C-4), 75.4 (C-1), 78.6 (C-3), 109.2 (C-2′ & 2″), 114.5 (C-8′),114.6 (C-5″), 114.7 (C-5″), 114.9 (C-8″), 123.2 (C-6′), 123.5 (C-6′), 126.7 (C-1′), 126.8 (C-1″), 145.5 (C-7′), 146.2 (C-7″), 146.7 (C-3′ & 3″), 148.0 (C-4″), 148.2 (C-4′), 166.1 (C-9″), 166.7 (C-9′), 173.4 (C-7); ESI-MS *m/z* 595 $[M+Na]^+$; HR-ESI-MS *m/z* 595.17836 $[M+Na]^+$ (calcd for $C_{29}H_{32}O_{12}Na$, 595.17860).

16-Pregnen-3,12,20-trione (**2**): amorphous powder; UV (MeOH) λ_{max} (log ε) 227 (3.65), 277 (sh, 2.69) nm; IR (KBr) υ_{max} 1711 (C=O), 1671 (C=O) cm^{-1}; ^{1}H-NMR ($CDCl_3$, 400 MHz) δ 1.08 (1H, ddd, *J* = 8.4, 8.0, 5.2 Hz, H-9α), 1.14 (3H, s, H-18), 1.28 (1H, ddd, *J* = 12.6, 10.4, 5.2 Hz, H-7), 1.36 (3H, s, H-19), 1.39 (1H, m, H-1), 1.42 (2H, m, H_2-2), 1.59 (1H, m, H-5),

1.68 (1H, td, J = 11.2, 6.8 Hz, H-14), 1.87 (2H, m, H-10β and 9β), 2.05 (1H, ddd, J = 11.2, 3.6, 3.6 Hz, H-8), 2.18 (1H, dd, J = 4.0, 2.0 Hz, H-4β), 2.21 (1H, dd, J = 6.8, 2.0 Hz, H-15β), 2.29 (2H, m, H-4α and H-11β), 2.34 (1H, s, H-21), 2.39 (1H, m, H-10α), 2.47 (1H, ddd, J = 16.8, 6.8, 3.2 Hz, H-15α), 2.68 (1H, t, J = 12.6 Hz, H-11α), 6.62 (1H, dd, J = 3.2, 2.0 Hz, H-16); ^{13}C-NMR ($CDCl_3$, 100 MHz) δ 11.2 (C-18), 16.6 (C-19), 27.4 (C-21), 28.6 (C-2), 30.8 (C-9), 31.7 (C-15), 33.3 (C-8), 36.5 (C-6), 37.7 (C-1), 37.8 (C-10), 38.1 (C-11), 44.3 (C-4), 46.3 (C-5), 55.8 (C-14), 56.3 (C-7), 61.1 (C-13), 142.2 (C-16), 150.6 (C-17), 196.2 (C-20), 209.1 (C-12), 210.7 (C-3); ESI-MS *m/z* 351 $[M+Na]^+$; HR-ESI-MS *m/z* 351.19291 $[M+Na]^+$ (calcd for $C_{21}H_{28}O_3Na$, 351.19307).

Deglucosylanodendrosin A (**3**): amorphous powder; UV (MeOH) λ_{max} (log ε) 212 (3.40), 262 (3.73) nm; IR (KBr) υ_{max} 3395 (OH), 1702 (C=O) cm^{-1}; ^{1}H-NMR ($CDCl_3$, 400 MHz) δ 1.78 (12H, s, H-4″, 4‴, 5″, & 5‴), 3.12 (1H, dd, J = 9.6, 2.8 Hz, H-3′), 3.36 (1H, dd, J = 9.6, 2.8 Hz, H-1), 3.37 (4H, d, J = 6.6 Hz, H_2-1″ & 1‴), 3.46 (3H, s, OMe-1′), 3.55 (3H, s, OMe-3′), 3.61 (1H, t, J = 9.8 Hz, H-5′), 4.03 (1H, dd, J = 9.8, 9.6 Hz, H-4′), 4.48 (1H, t, J = 2.8 Hz, H-2′), 5.31 (2H, mt, J = 6.6 Hz, H-2″& 2‴), 5.46 (1H, t, J = 9.6 Hz, H-6′), 5.90 (1H, br s, OH-4, D_2O exchangable), 7.73 (2H, s, H-2 & 6); ^{13}C-NMR ($CDCl_3$, 100 MHz) δ 17.9 (C-5″ & 5‴), 25.8 (C-4″ & 4‴), 29.6 (H-1″ & H-1‴), 57.8 (OMe-3′), 58.8 (OMe-1′), 65.4 (C-2′), 72.4 (C-4′), 73.5 (C-5′), 74.3 (C-6′), 79.9 (C-1′), 80.8 (C-3′), 121.3 (C-2″ &2‴), 121.5 (C-1), 127.1 (C-3 & 5), 130.1 (C-2 & 6), 135.1 (C-3″ & 3‴), 157.5 (C-4), 167.1 (C-7); ESI-MS *m/z* 487 $[M+Na]^+$; HR-ESI-MS *m/z* 487.23009 $[M+Na]^+$ (calcd for $C_{25}H_{36}O_8Na$, 487.23024).

Anodendrosin H (**4**): amorphous powder; UV (MeOH) λ_{max} (log ε) 245 (4.56), 280 (sh, 3.77) nm; IR (KBr) υ_{max} 3404 (OH), 1710 (C=O) cm^{-1}; ^{1}H-NMR ($CDCl_3$, 500 MHz) δ 1.44 (6H, s, Me_2-2), 1.73 (6H, s, H-4″ and H-5″), 3.12 (1H, dd, *J* = 9.5, 3.0 Hz, H-1′), 3.28 (2H, d, *J* = 7.5 Hz, H-1″), 3.35 (1H, dd, *J* = 9.5, 3.0, H-1′), 3.46 (3H, s, OMe-3′), 3.55 (1H, s, OMe-1′), 3.60 (1H, dd, *J* = 10.0, 9.5 Hz, H-5′), 4.03 (1H, t, *J* = 9.5 Hz, H-4′), 4.48 (1H, t, *J* = 3.0 Hz, H-2′), 5.26 (1H, br t, *J* = 7.5 Hz, H-2″), 5.47 (1H, dd, *J* = 10.0, 9.5, H-6′), 5.65 (1H, d, *J* = 9.8, H-3), 6.34 (1H, d, *J* = 9.8, H-4), 7.57 (1H, d, *J* = 2.0, H-7), 7.72 (1H, d, *J* = 2.0, H-5).

4-Hydroxy-3-prenylbenzoic acid (**5**): white needles; m.p. 101~105 ℃; UV (MeOH) λ_{max} (log ε) 201 (4.61), 214 (sh, 4.21), 257 (4.00) nm; IR (KBr) υ_{max} 3199 (OH), 1679 (C=O) cm^{-1}; ^{1}H-NMR ($CDCl_3$, 500 MHz) δ 1.80 (6H, s, H-4′ and H-5′), 3.41 (2H, d, *J* = 7.0 Hz, H-1′), 5.33 (1H, br t, *J* = 7.0 Hz, H-2′), 6.85 (1H, d, *J* = 8.8, H-5), 7.89 (1H, s, H-2), 7.90 (1H, d, *J* = 8.8, H-6).

Gelseminic acid (**6**): amorphous needles; m.p. 203~206 ℃; UV (MeOH) λ_{max} (log ε) 228 (4.32), 260 (3.31), 292 (3.32), 345 (4.16) nm; IR (KBr) υ_{max} 3334 (OH), 1711 (C=O) cm^{-1}; ^{1}H-NMR ($CDCl_3$, 400 MHz) δ 3.96 (3H, s, OMe-6), 6.13 (1H, br s, OH-1, D_2O exchangable), 6.27 (1H, d, *J* = 9.6 Hz, H-3), 6.85 (1H, s, H-8), 6.92 (1H, s, H-5), 7.60 (1H, d, *J* = 9.6, H-4).

MS, IR, and NMR spectrum of isolated compounds

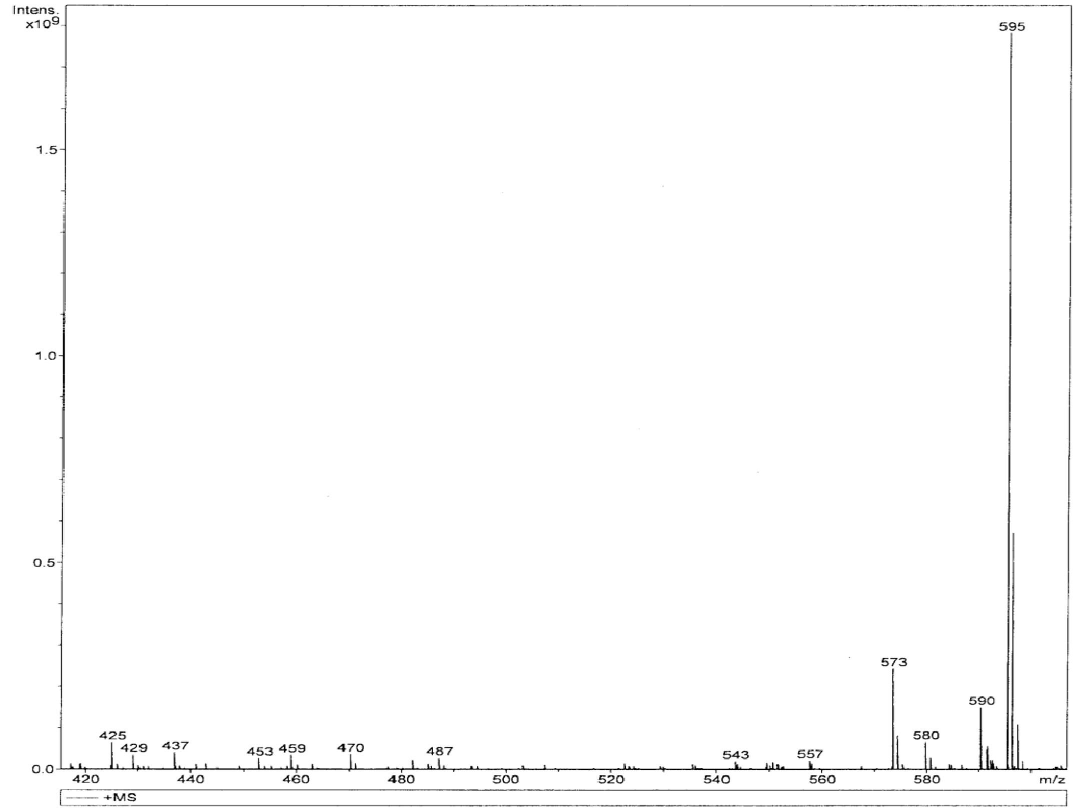

Figure S1. ESI-MS spectrum of Methyl 4,5-*O*-feruloyl-3-methoxyquinate (**1**).

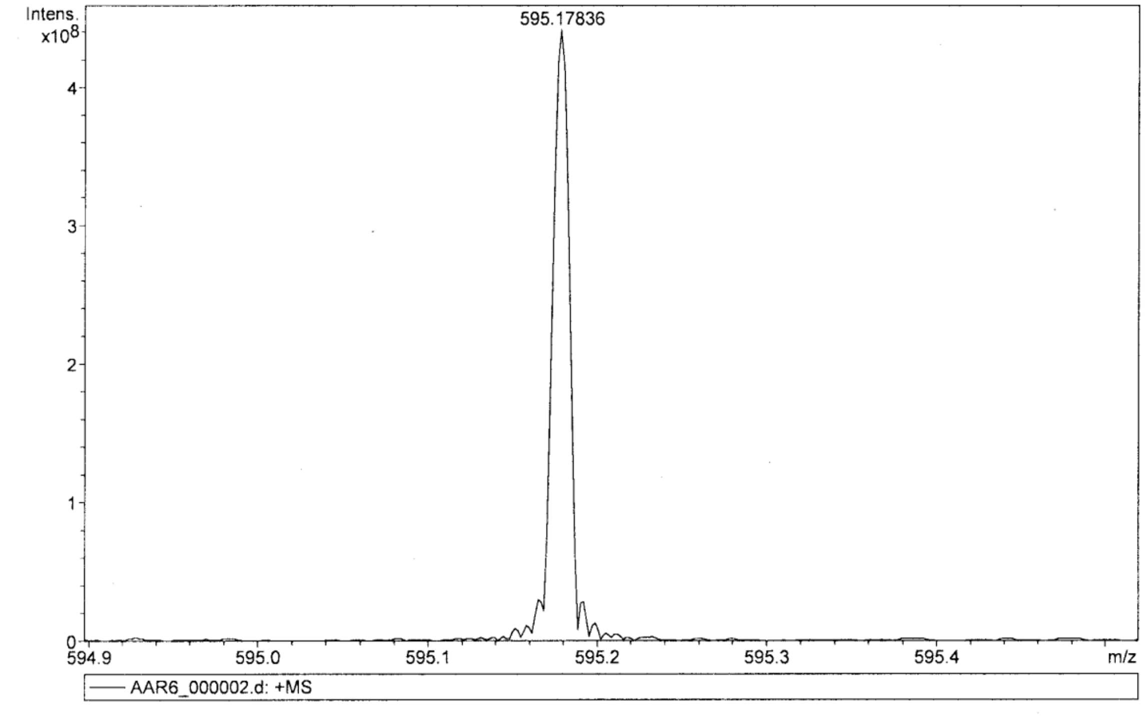

Meas. m/z	#	Formula	Score	m/z	err [mDa]	err [ppm]	mSigma	rdb	e⁻ Conf	N-Rule
595.17836	1	C 29 H 32 Na O 12	100.00	595.17860	0.23	0.39	9.7	13.5	even	ok

Figure S2. HR-ESI-MS spectrum of Methyl 4,5-*O*-feruloyl-3-methoxyquinate (**1**).

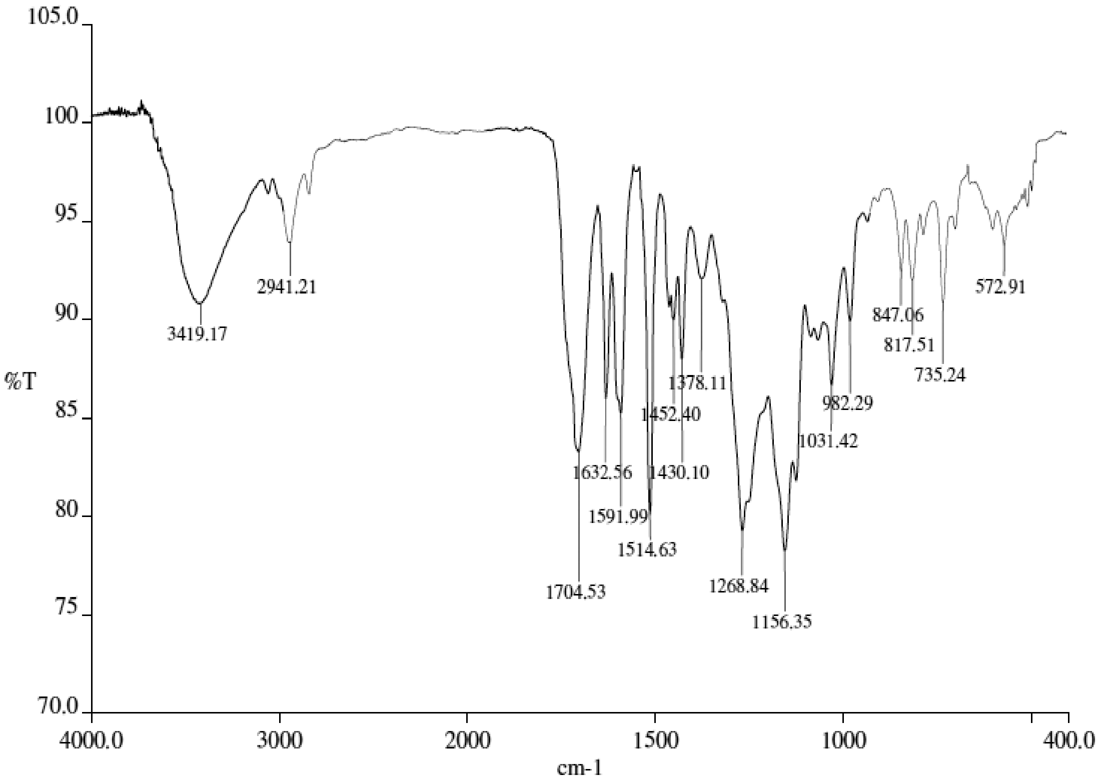

Figure S3. IR spectrum of Methyl 4,5-*O*-feruloyl-3-methoxyquinate (**1**).

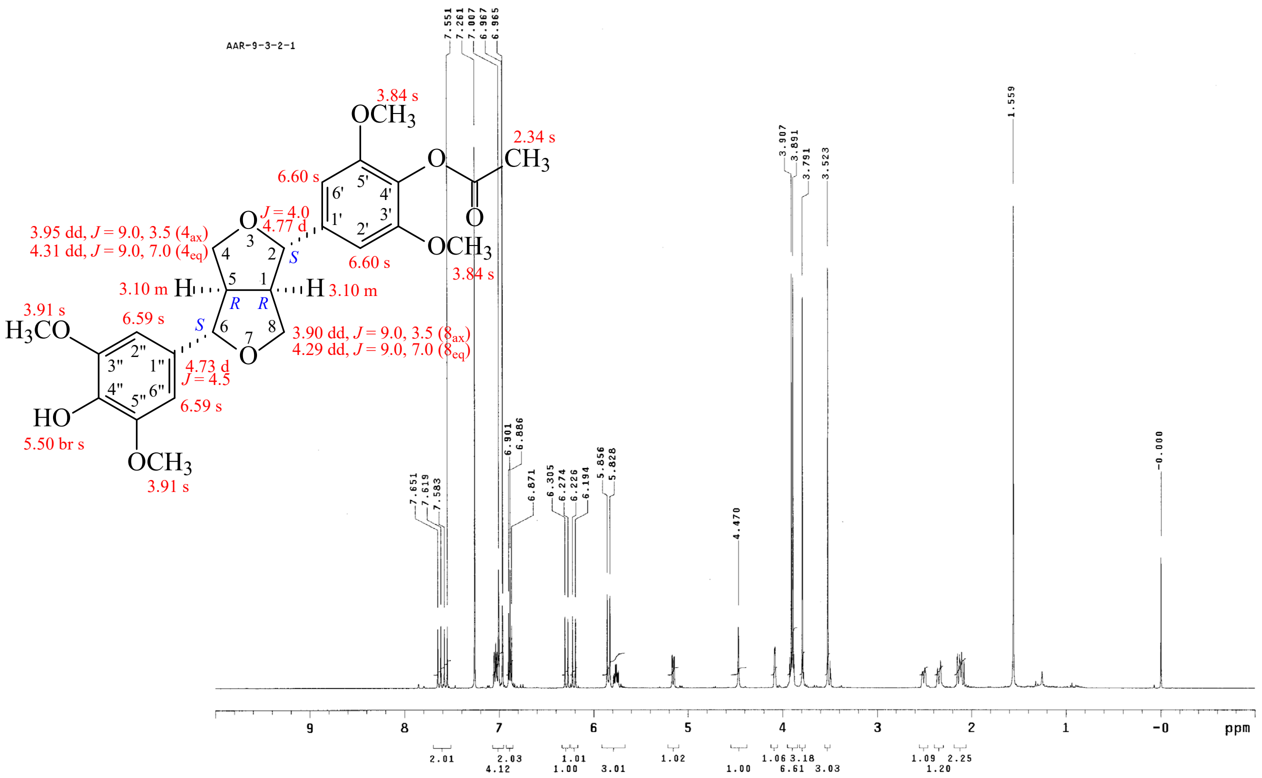

Figure S4. ^{1}H-NMR spectrum of Methyl 4,5-*O*-feruloyl-3-methoxyquinate (**1**).

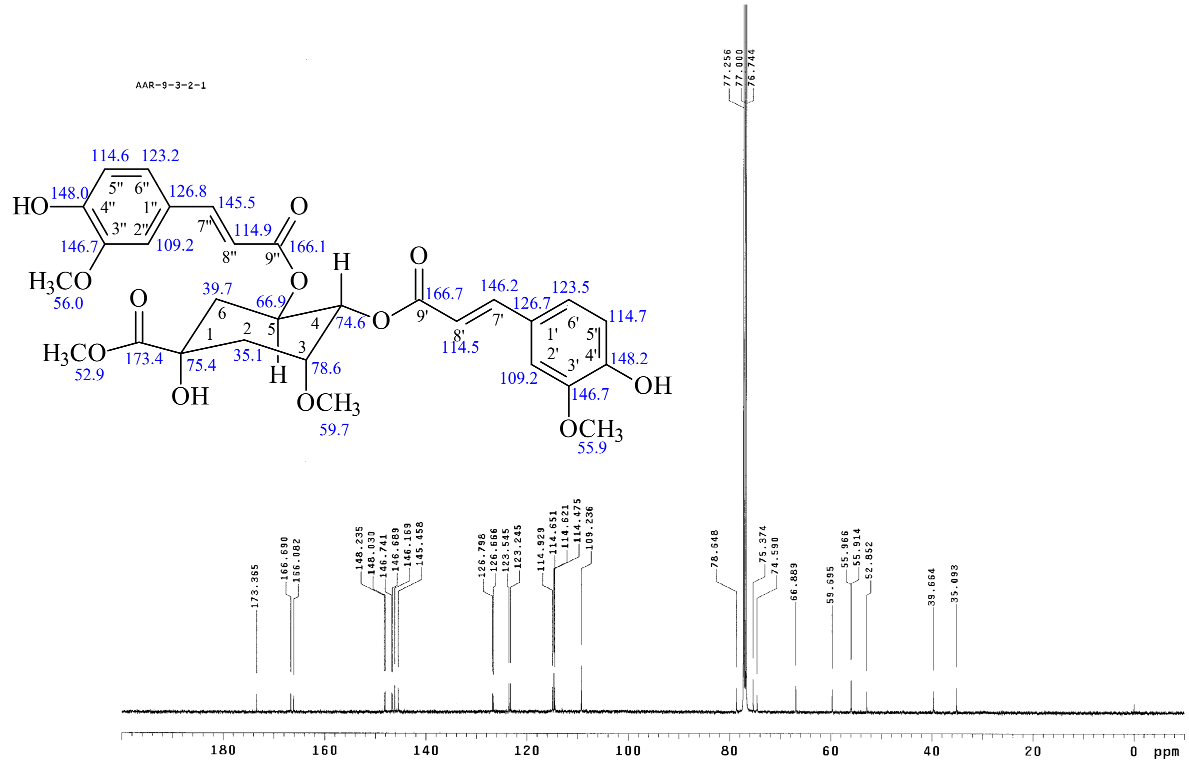

Figure S5. ^{13}C-NMR spectrum of Methyl 4,5-*O*-feruloyl-3-methoxyquinate (**1**).

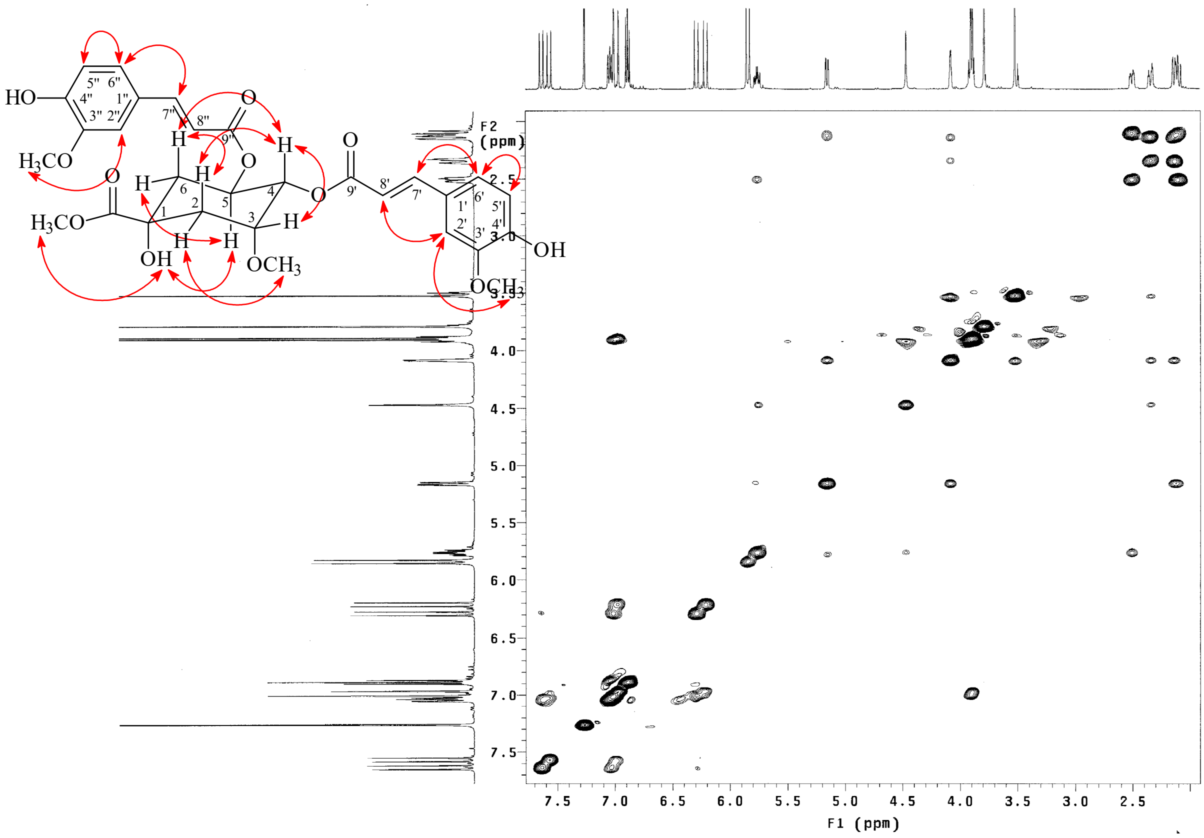

Figure S6. NOESY spectrum of Methyl 4,5-*O*-feruloyl-3-methoxyquinate (**1**).

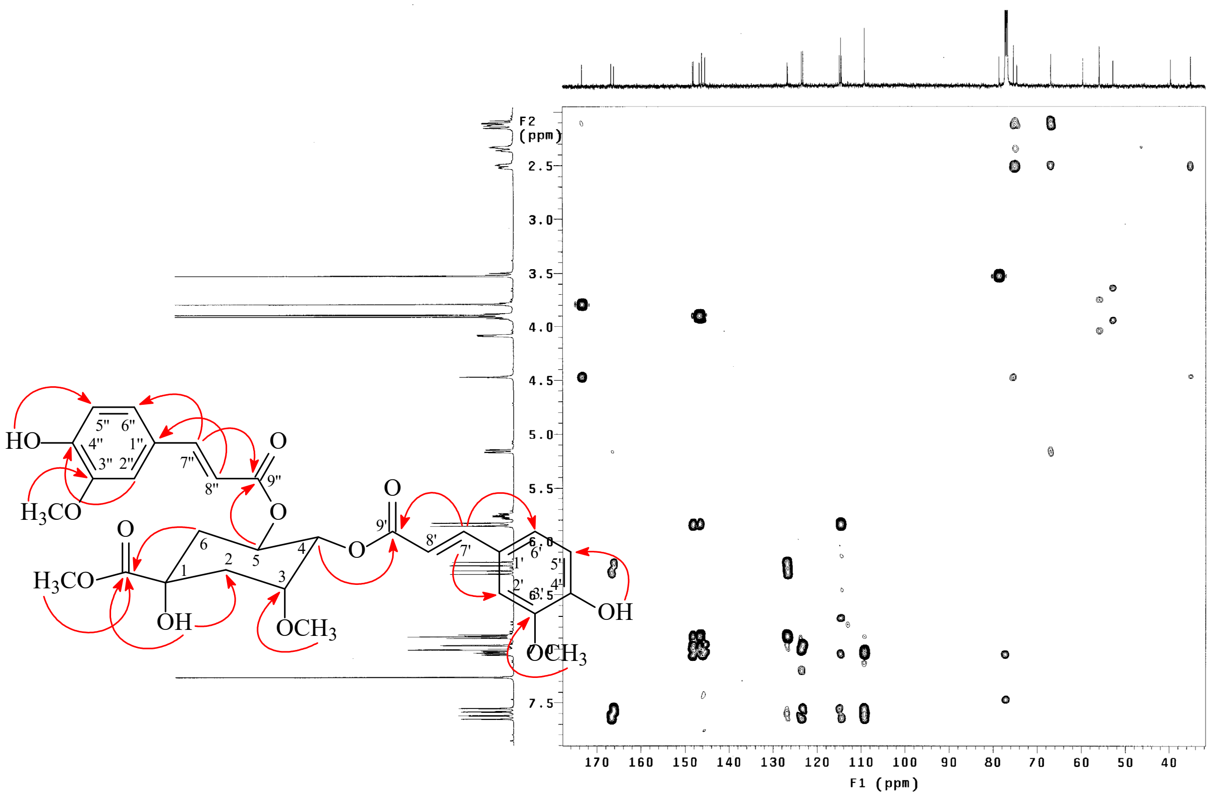

Figure S7. HMBC spectrum of Methyl 4,5-*O*-feruloyl-3-methoxyquinate (**1**).

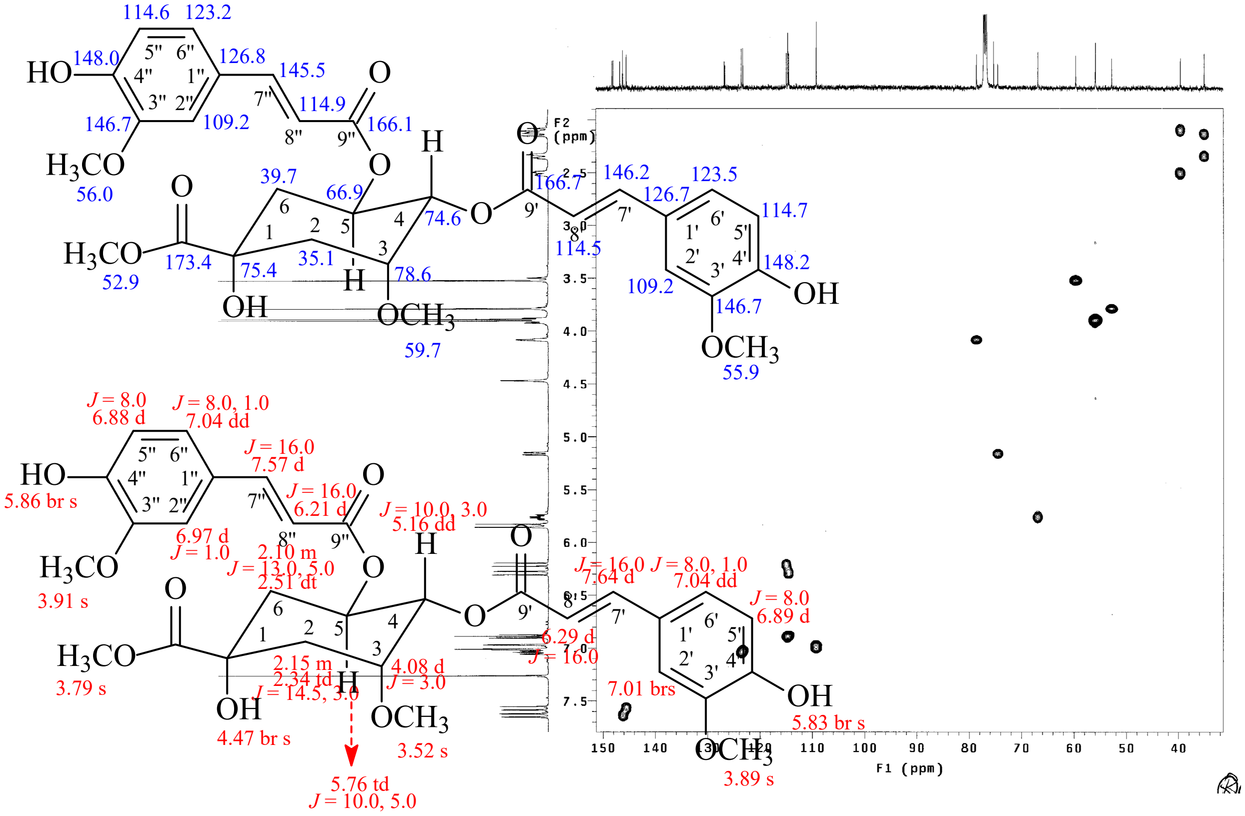

Figure S8. HSQC spectrum of Methyl 4,5-*O*-feruloyl-3-methoxyquinate (**1**).

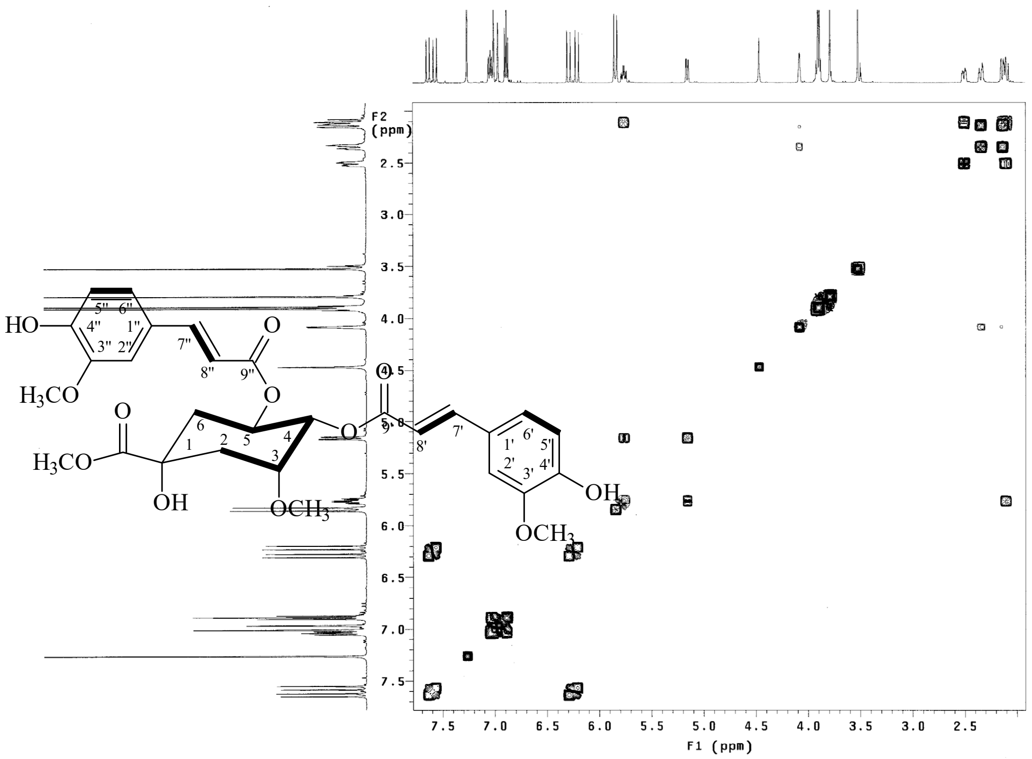

Figure S9. ^{1}H-^{1}H COSY spectrum of Methyl 4,5-*O*-feruloyl-3-methoxyquinate (**1**).

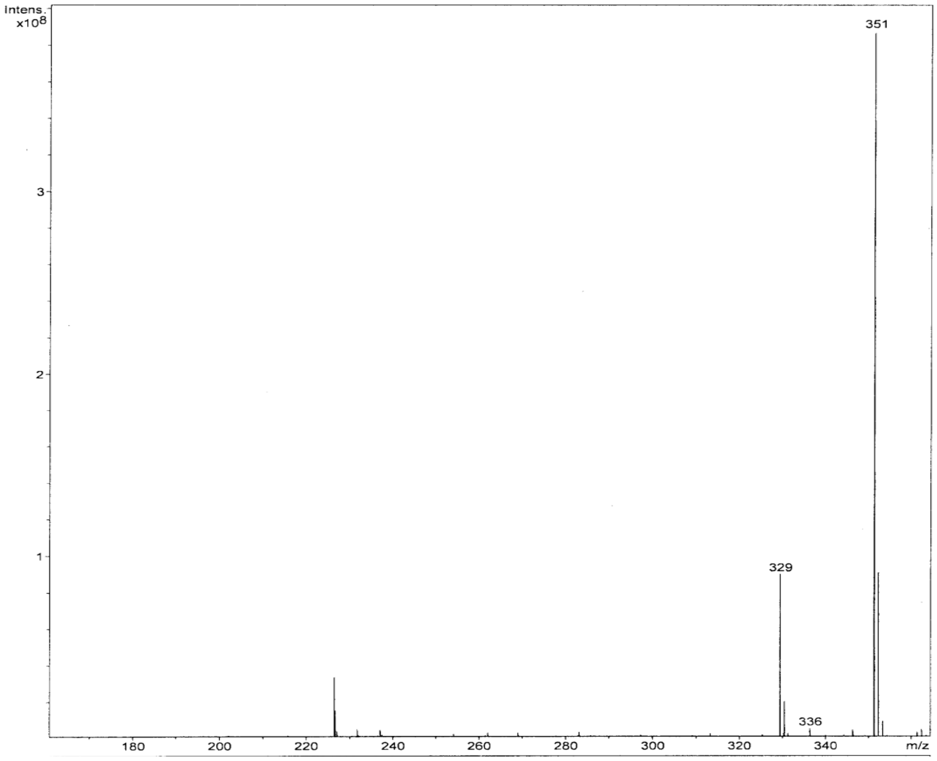

Figure S10. ESI-MS spectrum of 16-Pregnen-3,12,20-trione (**2**).

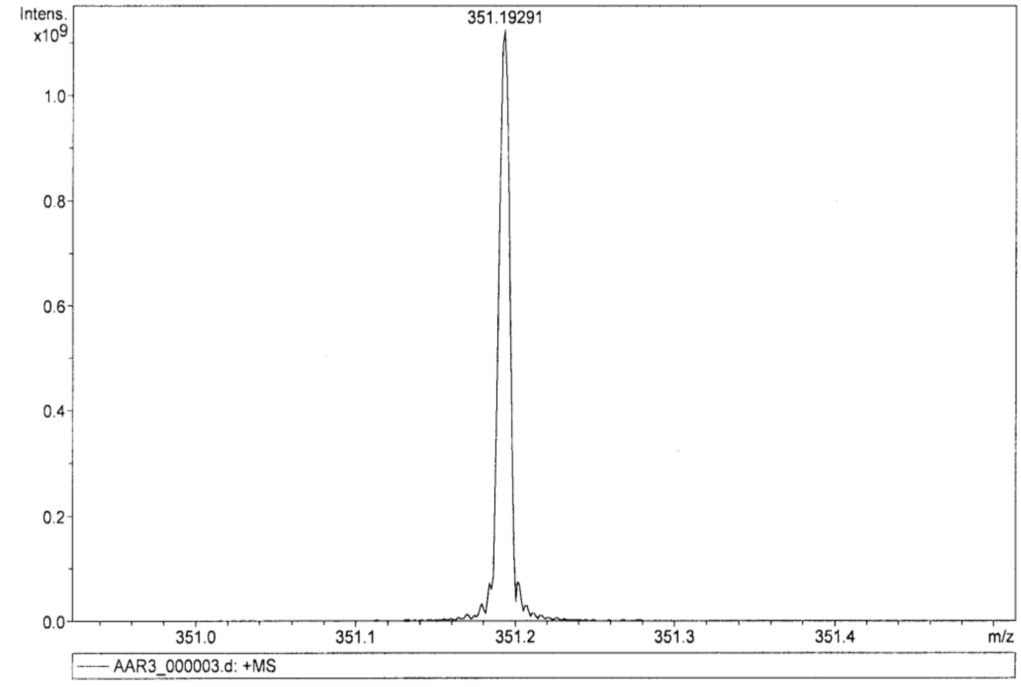

Meas. m/z	#	Formula	Score	m/z	err [mDa]	err [ppm]	mSigma	rdb	e⁻ Conf	N-Rule
351.19291	1	C 21 H 28 Na O 3	100.00	351.19307	0.15	0.43	3.8	7.5	even	ok

Figure S11. HR-ESI-MS spectrum of 16-Pregnen-3,12,20-trione (**2**).

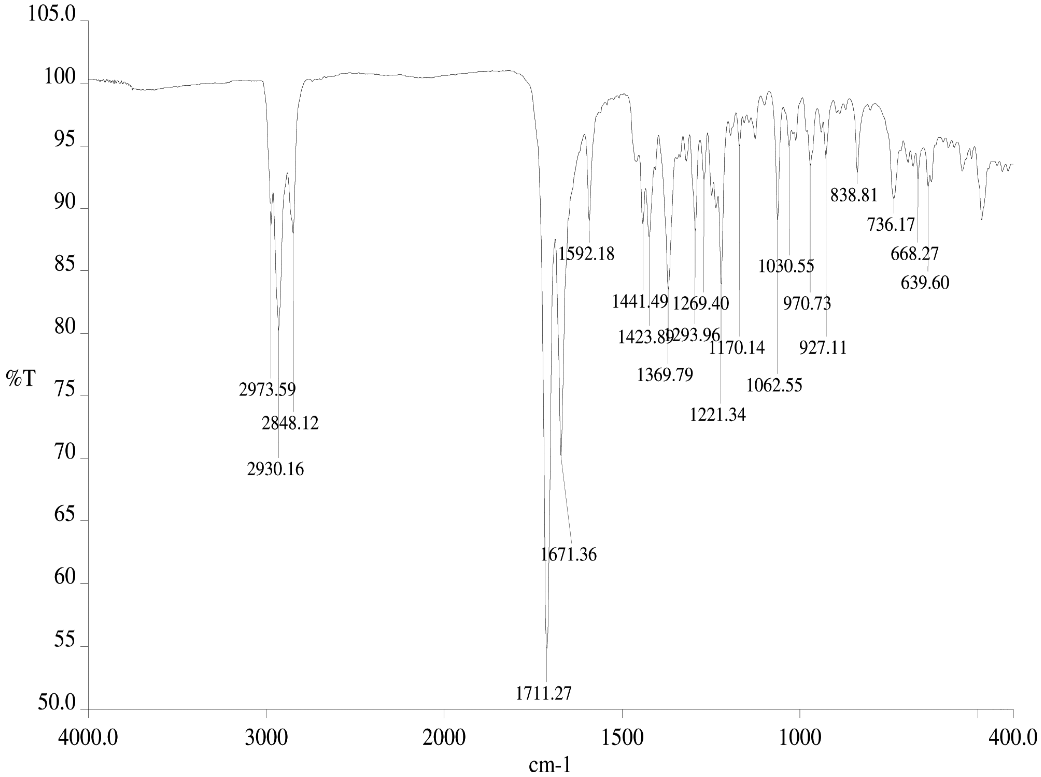

Figure S12. IR spectrum of 16-Pregnen-3,12,20-trione (**2**).

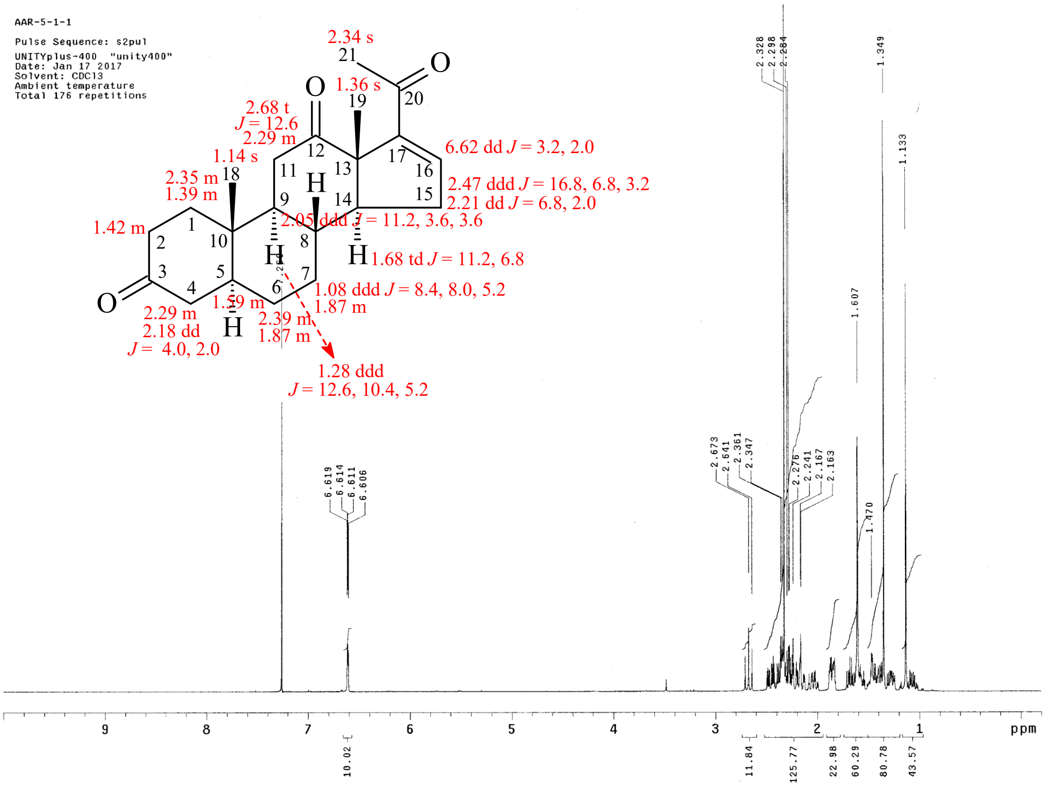

Figure S13. ^{1}H-NMR spectrum of 16-Pregnen-3,12,20-trione (**2**).

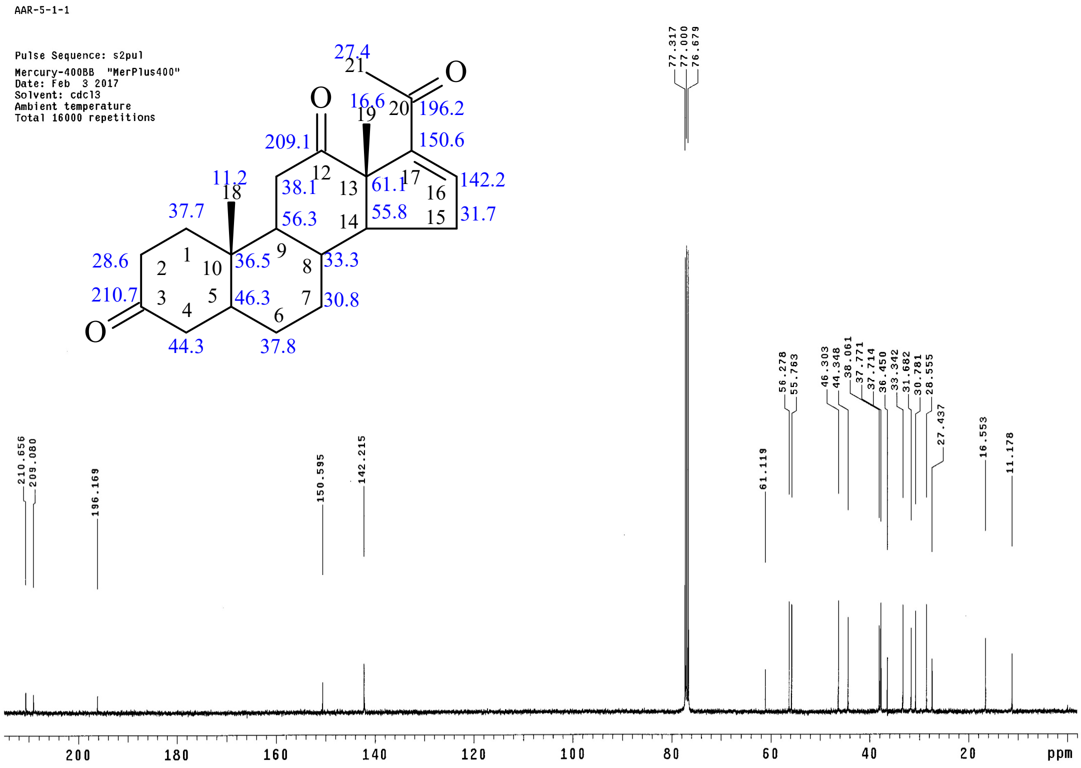

Figure S14. ^{13}C-NMR spectrum of 16-Pregnen-3,12,20-trione (**2**).

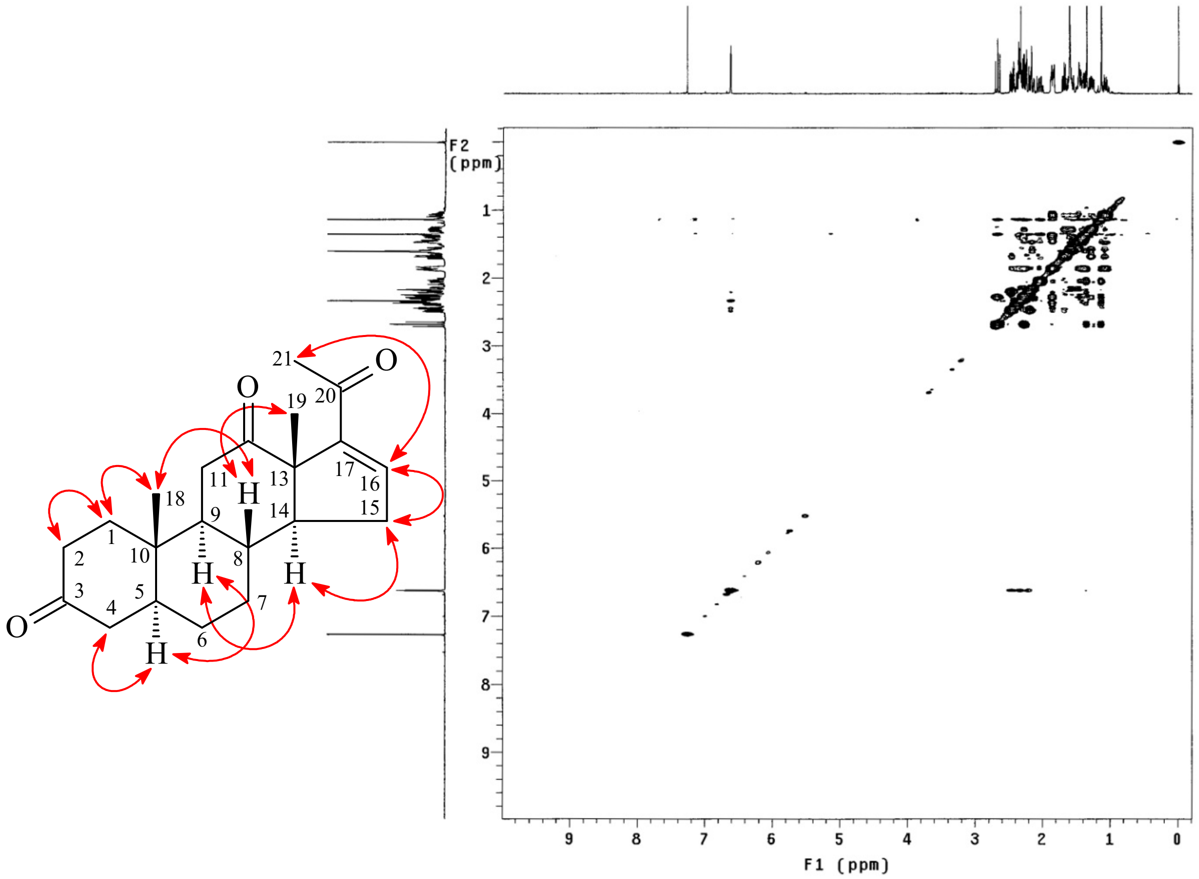

Figure S15. NOESY spectrum of 16-Pregnen-3,12,20-trione (**2**).

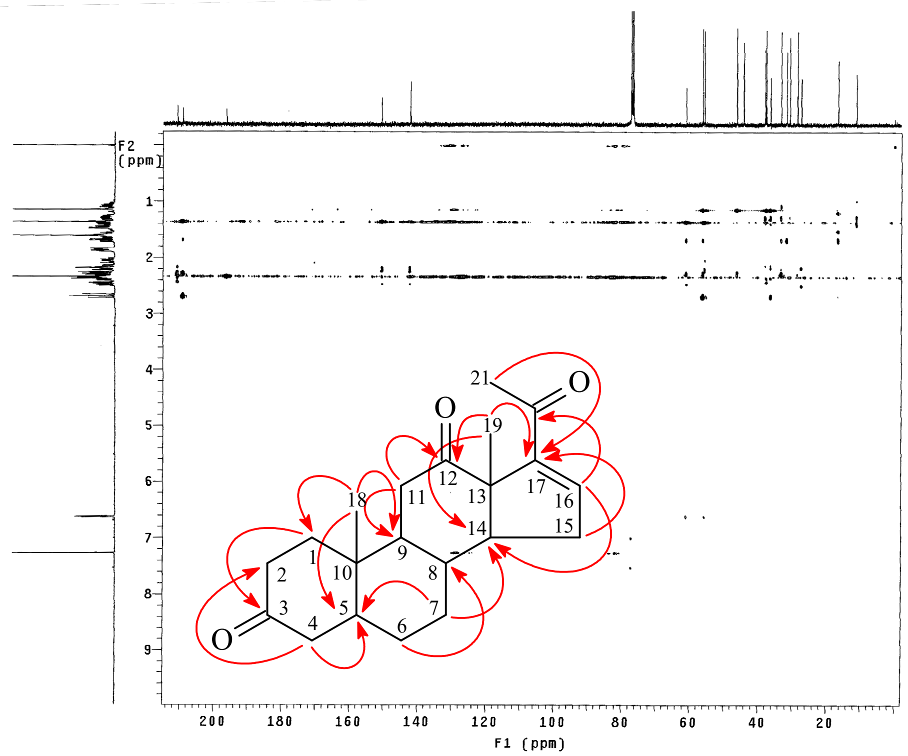

Figure S16. HMBC spectrum of 16-Pregnen-3,12,20-trione (**2**).

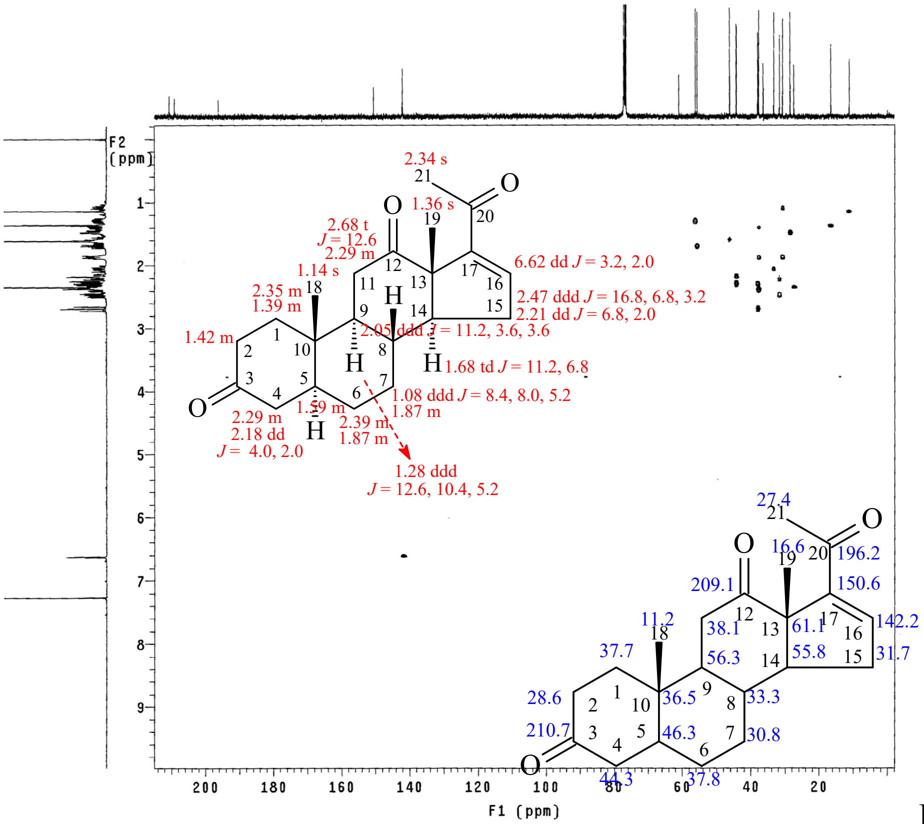

Figure S17. HSQC spectrum of 16-Pregnen-3,12,20-trione (**2**).

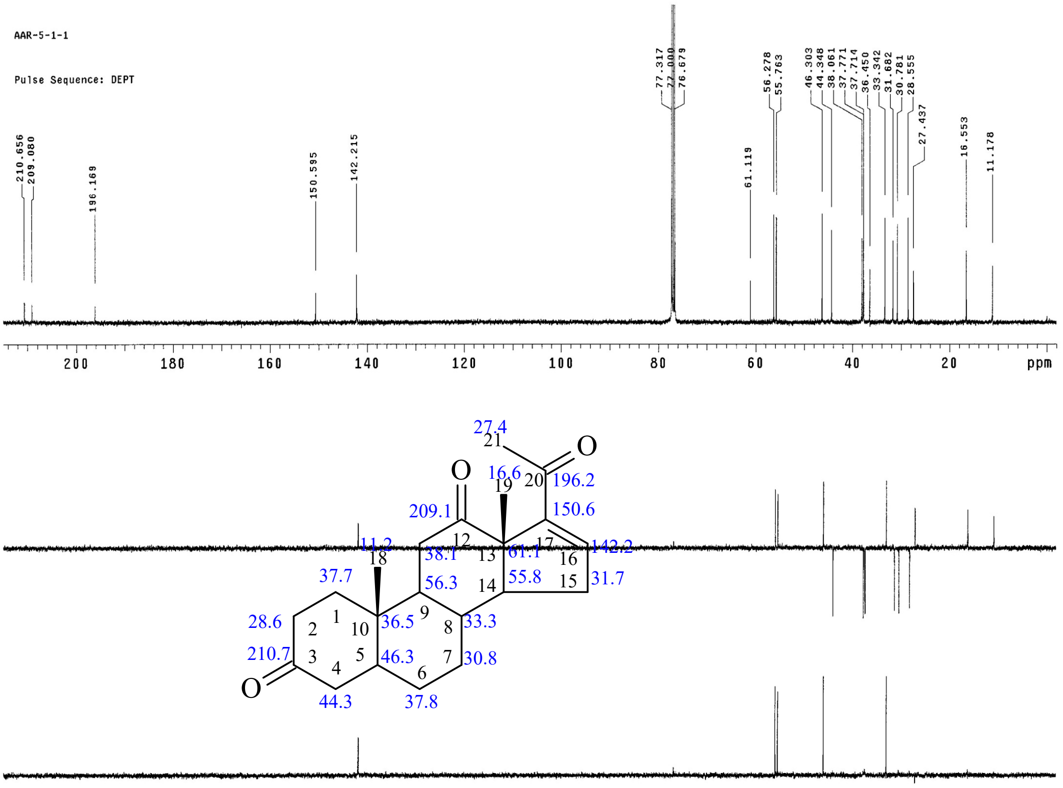

Figure S18. DEPT spectrum of 16-Pregnen-3,12,20-trione (**2**).

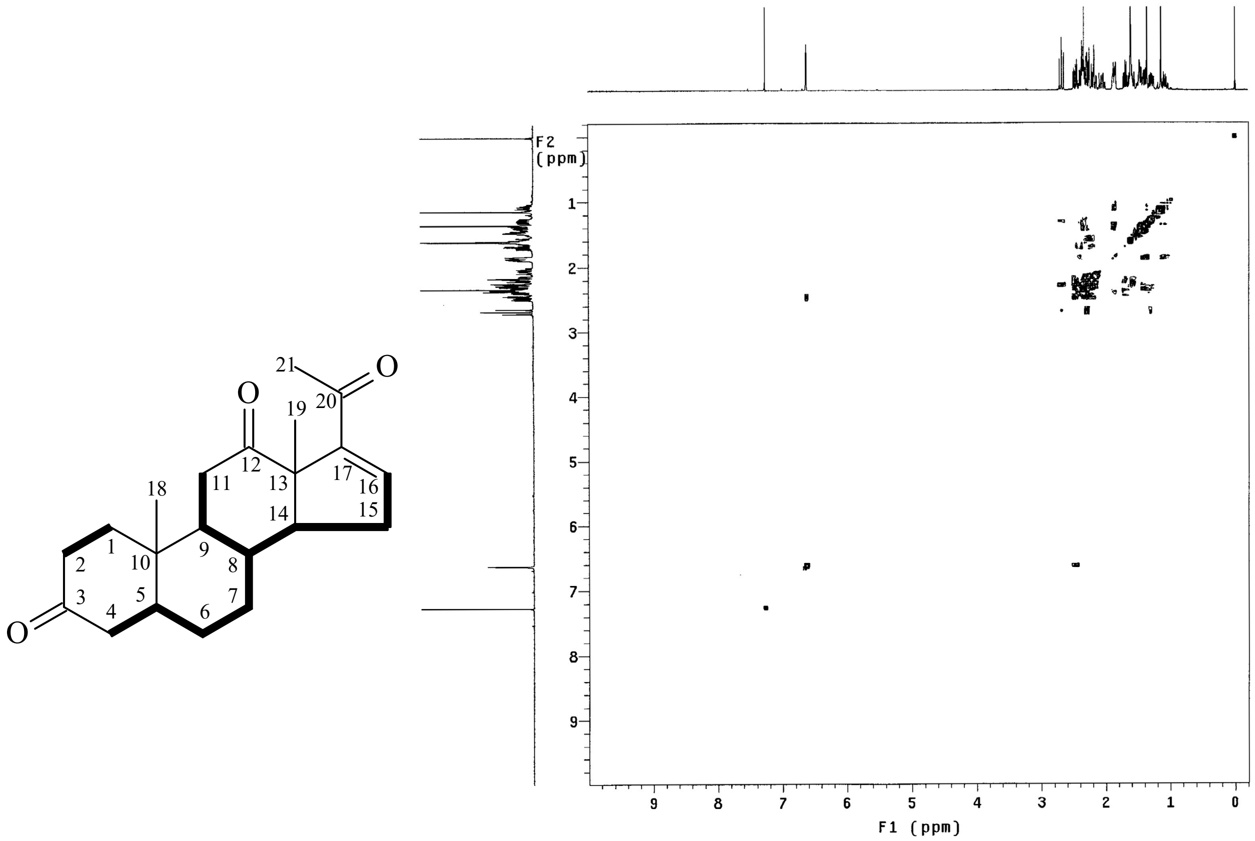

Figure S19. ^{1}H-^{1}H COSY spectrum of 16-Pregnen-3,12,20-trione (**2**).

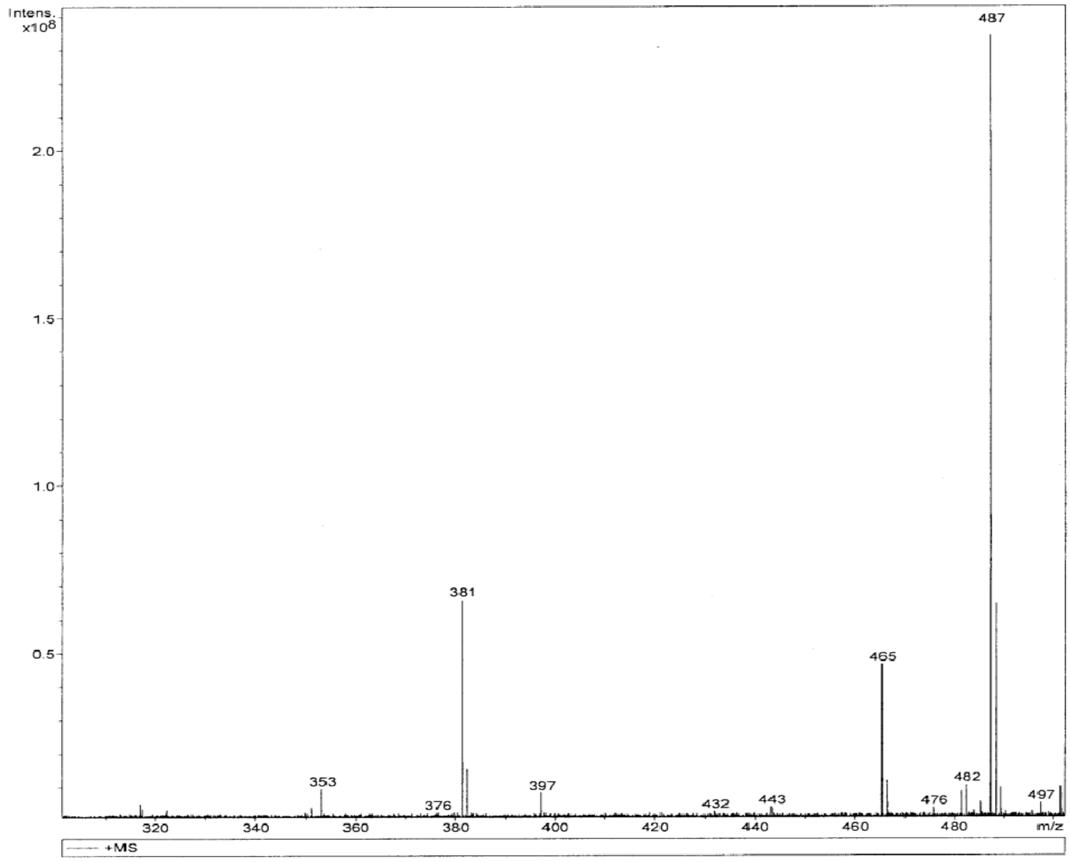

Figure S20. ESI-MS spectrum of Deglucosylanodendrosin A (**3**).

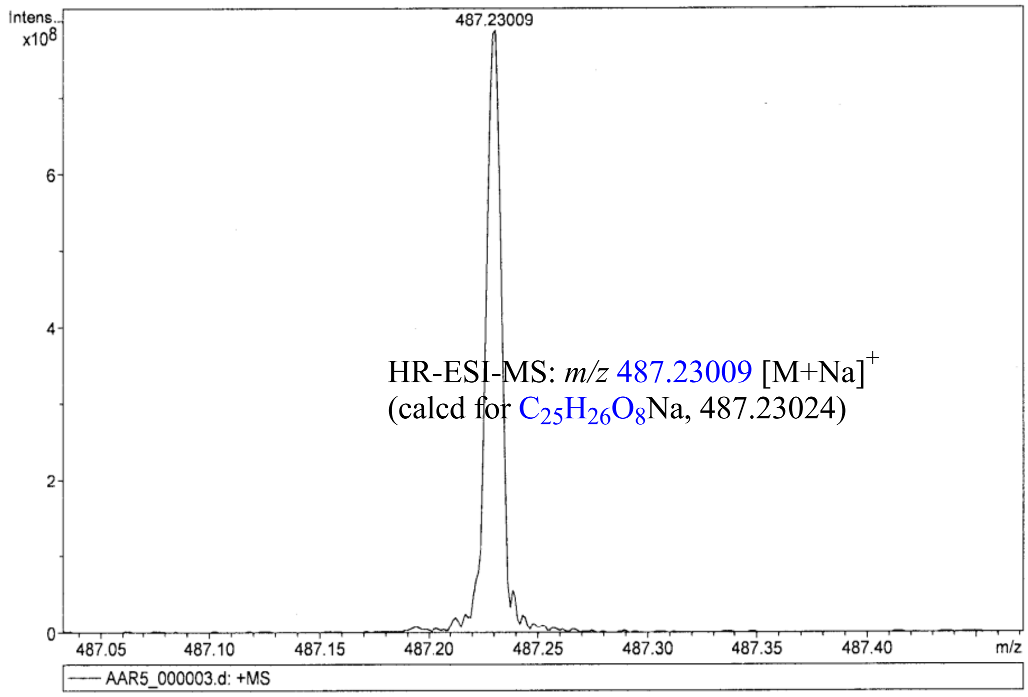

Meas. m/z	#	Formula	Score	m/z	err [mDa]	err [ppm]	mSigma	rdb	e⁻ Conf	N-Rule
487.23009	1	C 25 H 36 Na O 8	100.00	487.23024	0.15	0.31	7.7	7.5	even	ok

Figure S21. HR-ESI-MS spectrum of Deglucosylanodendrosin A (**3**).

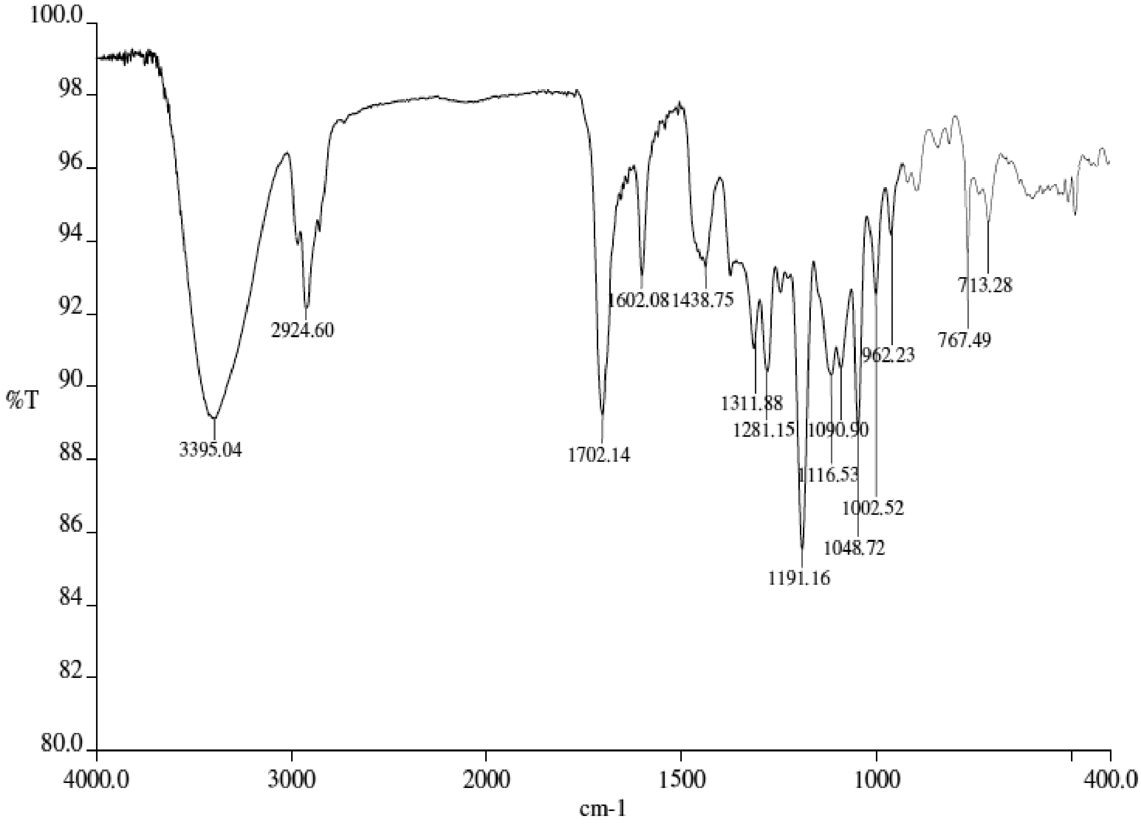

Figure S22. IR spectrum of Deglucosylanodendrosin A (**3**).

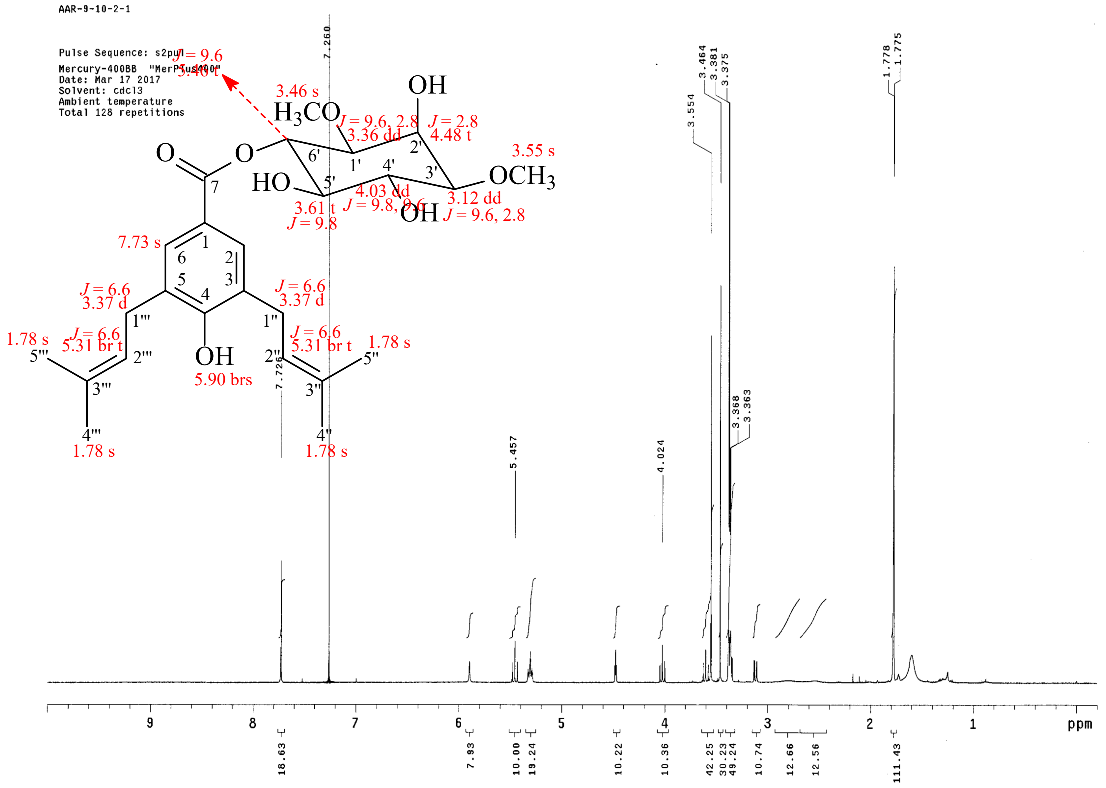

Figure S23. ^{1}H-NMR spectrum of Deglucosylanodendrosin A (**3**).

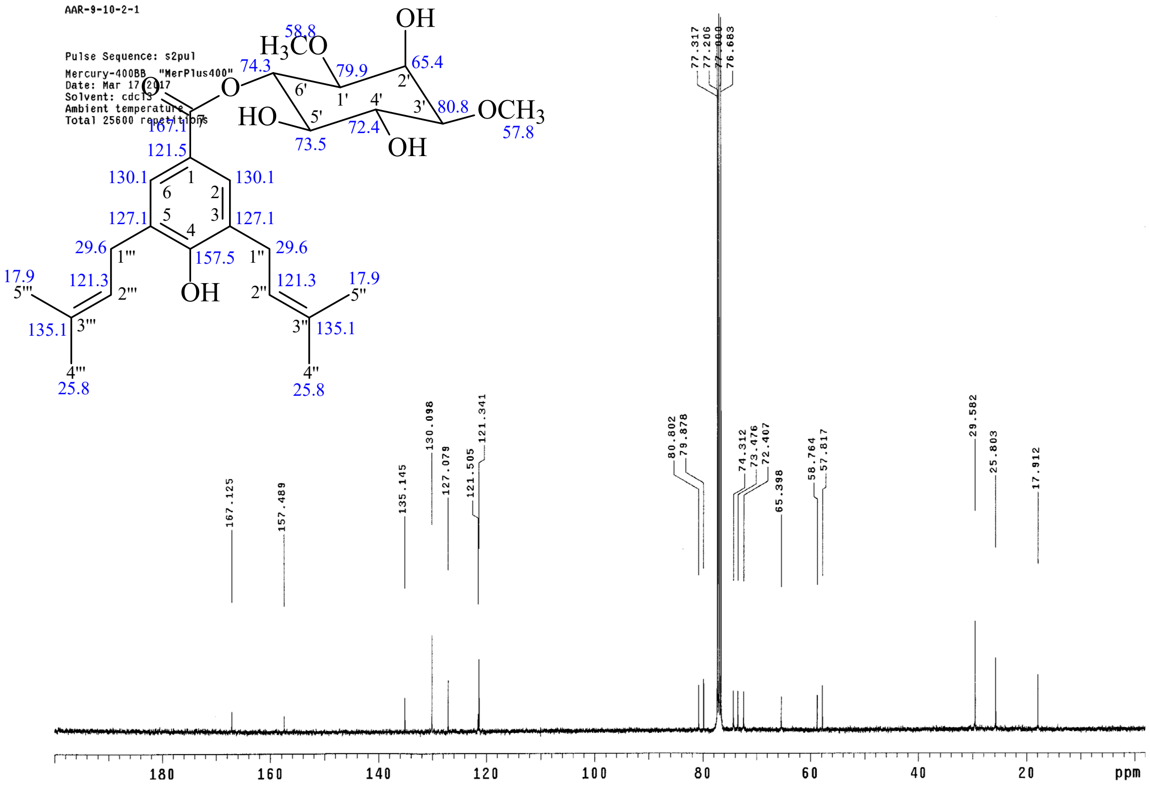

Figure S24. ^{13}C-NMR spectrum of Deglucosylanodendrosin A (**3**).

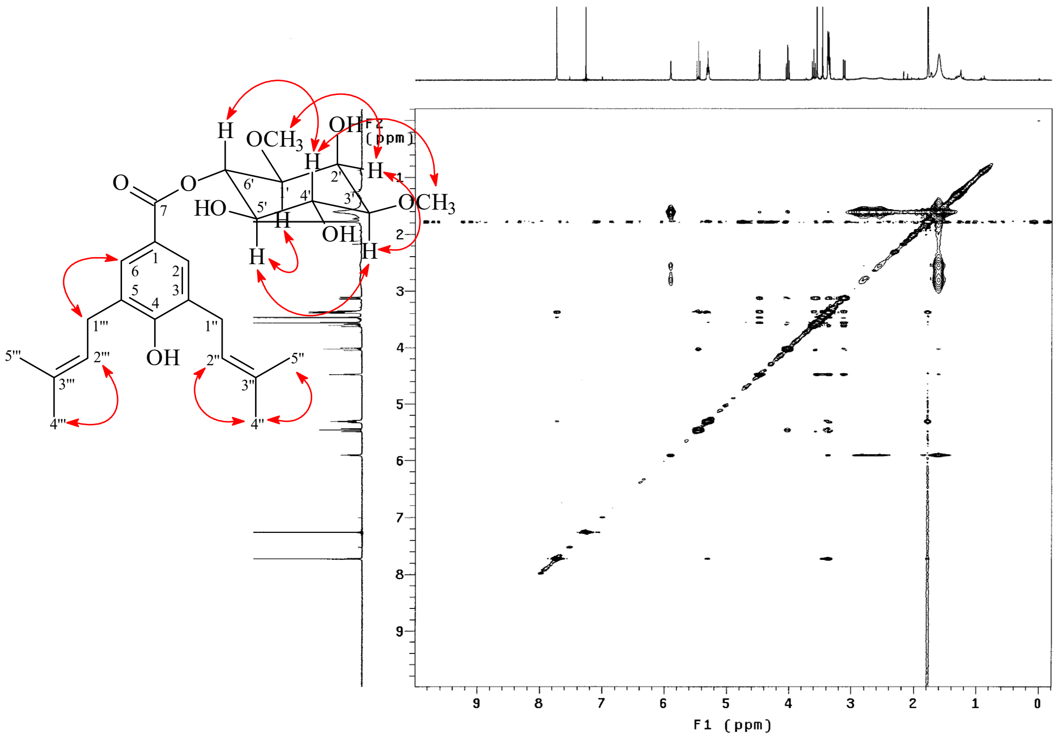

Figure S25. NOESY spectrum of Deglucosylanodendrosin A (**3**).

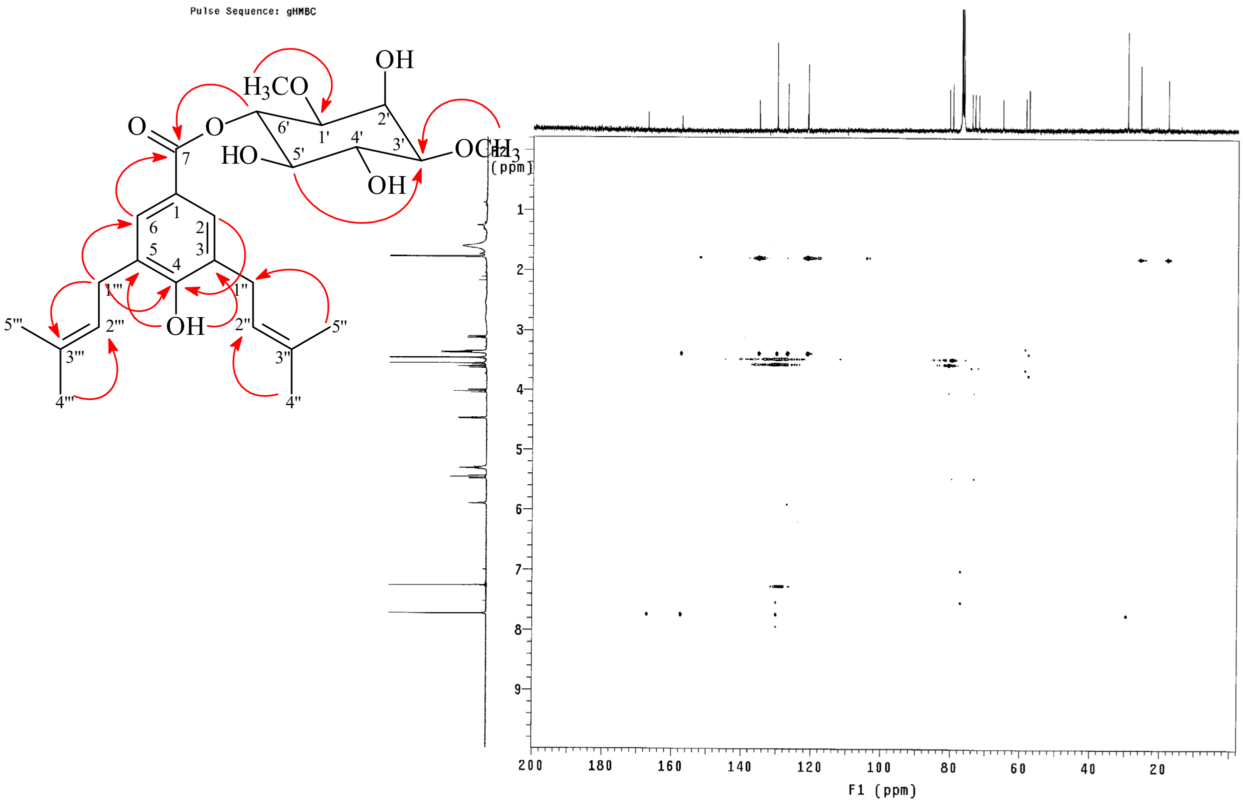

Figure S26. HMBC spectrum of Deglucosylanodendrosin A (**3**).

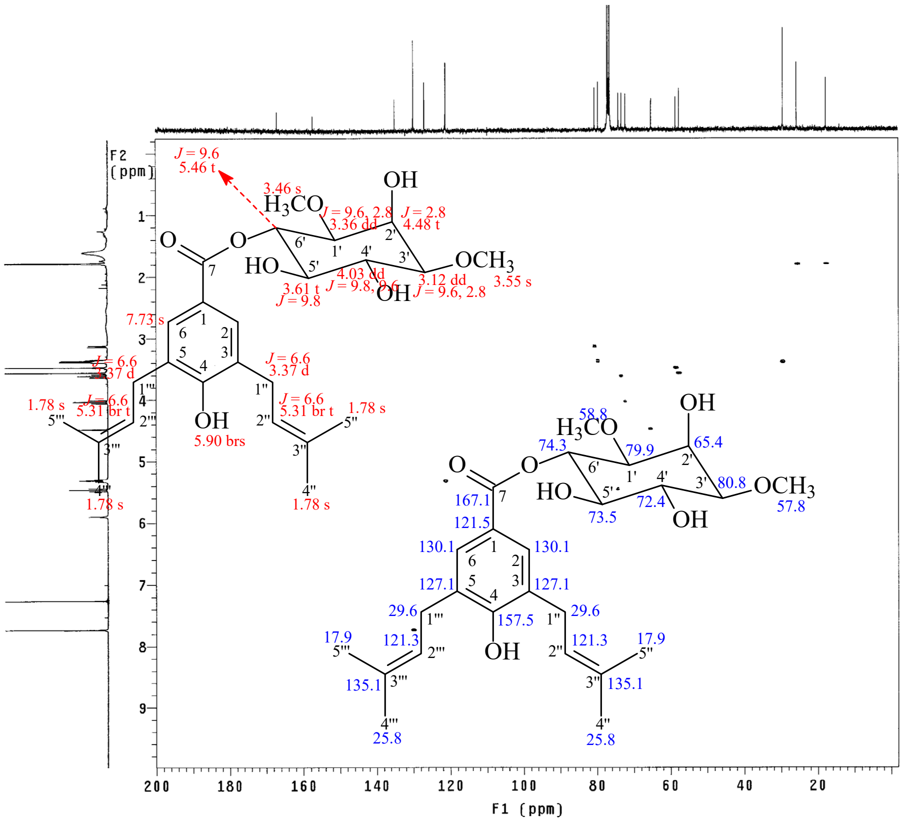

Figure S27. HSQC spectrum of Deglucosylanodendrosin A (**3**).

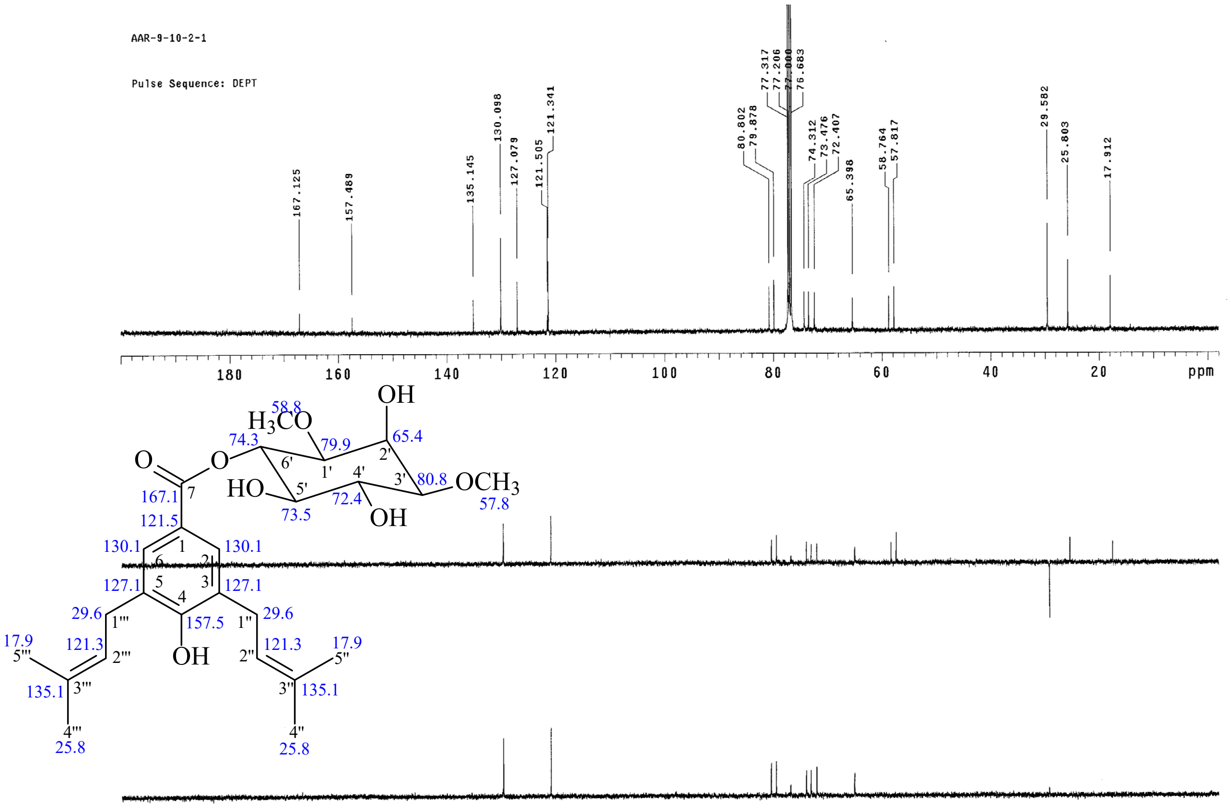

Figure S28. DEPT spectrum of Deglucosylanodendrosin A (**3**).

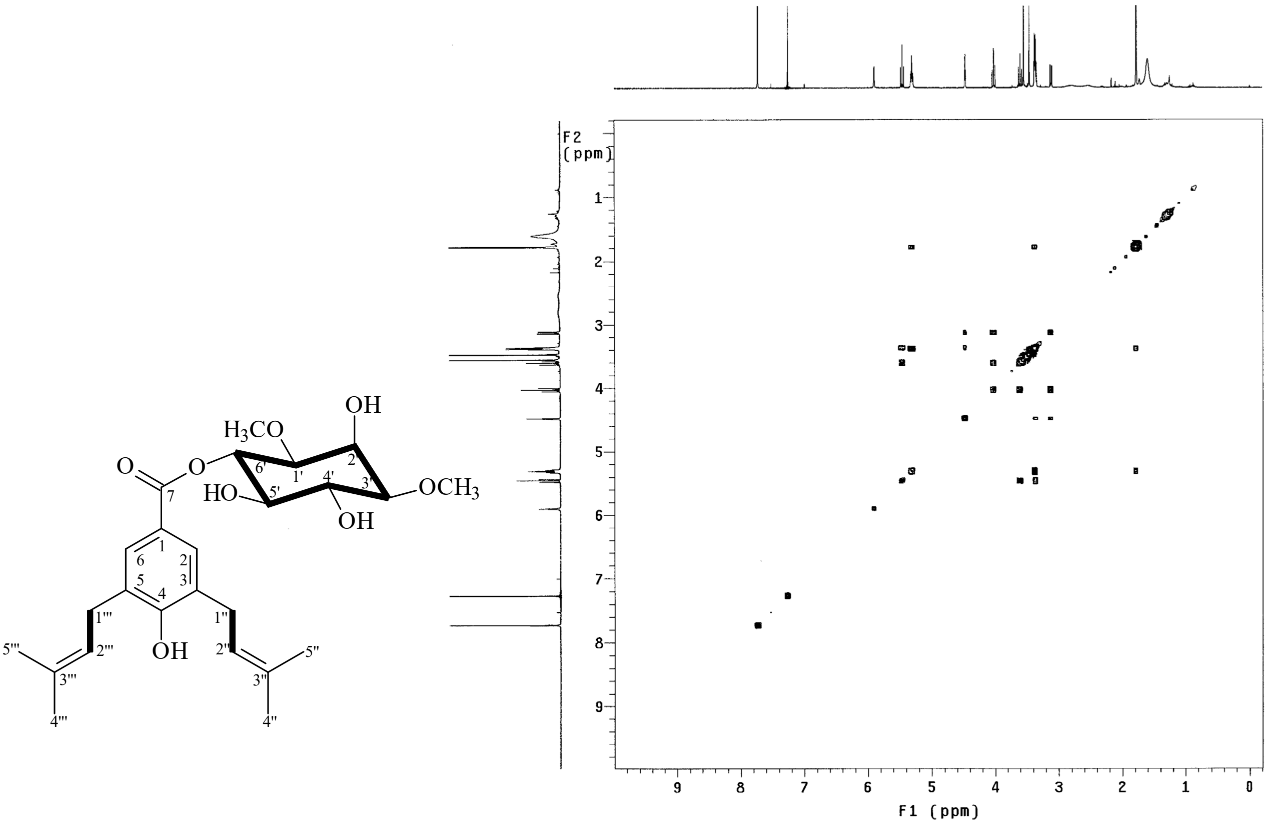

Figure S29. ^{1}H-^{1}H COSY spectrum of Deglucosylanodendrosin A (**3**).

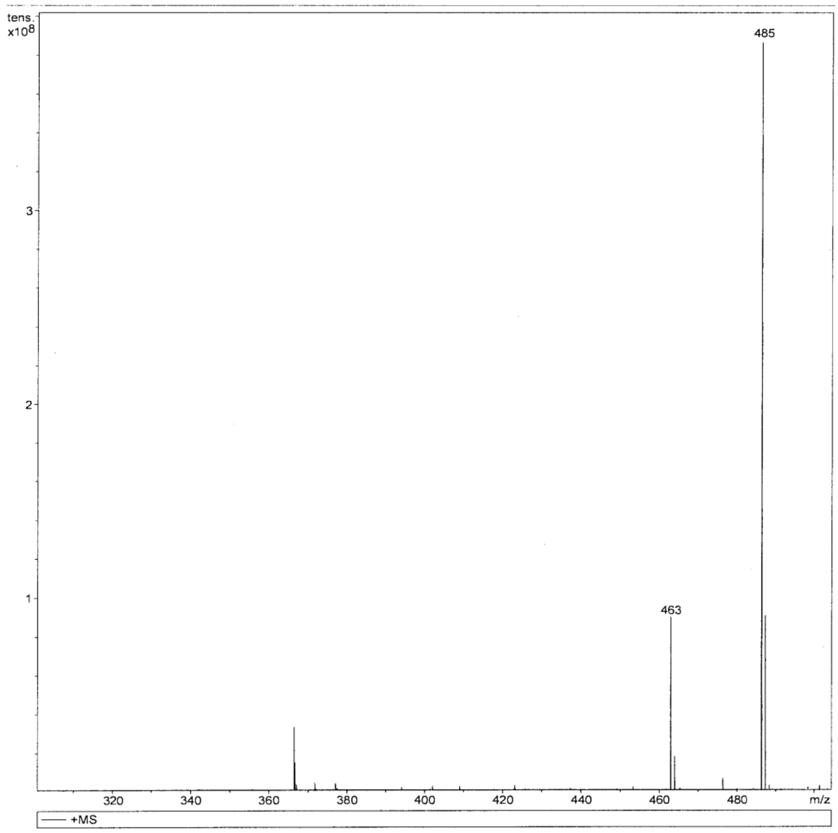

Figure S30. ESI-MS spectrum of Anodendrosin H (**4**).

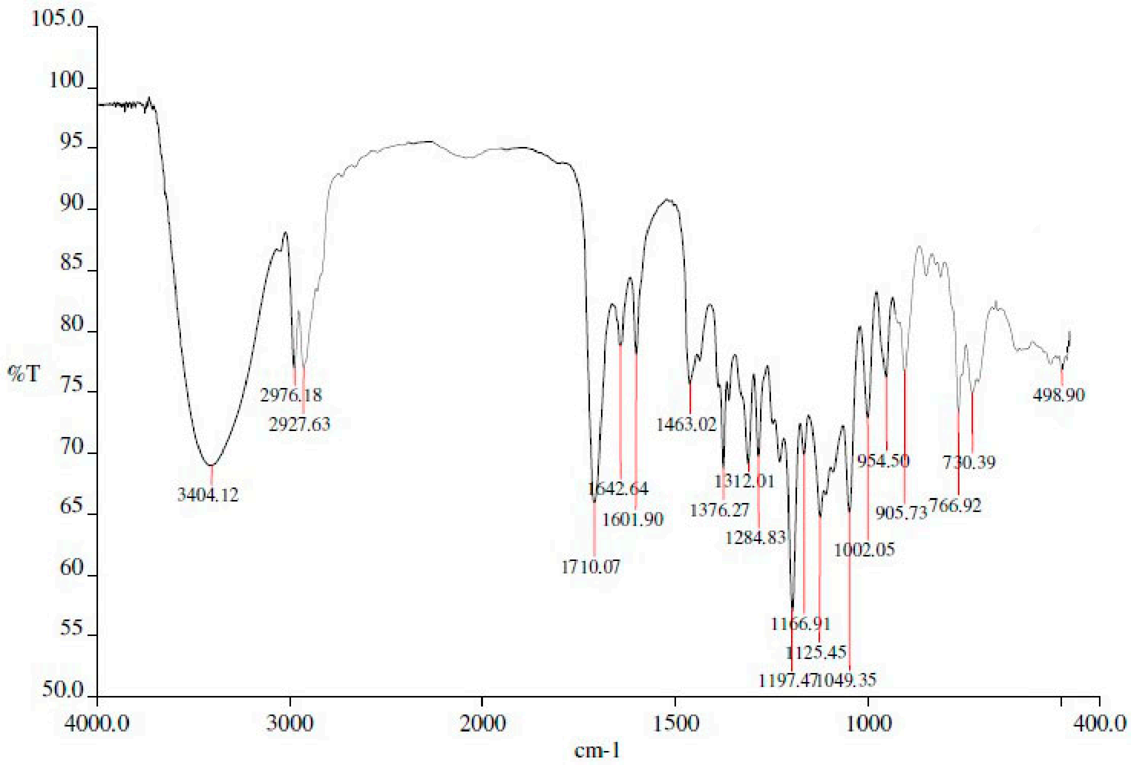

Figure S31. IR spectrum of Anodendrosin H (**4**).

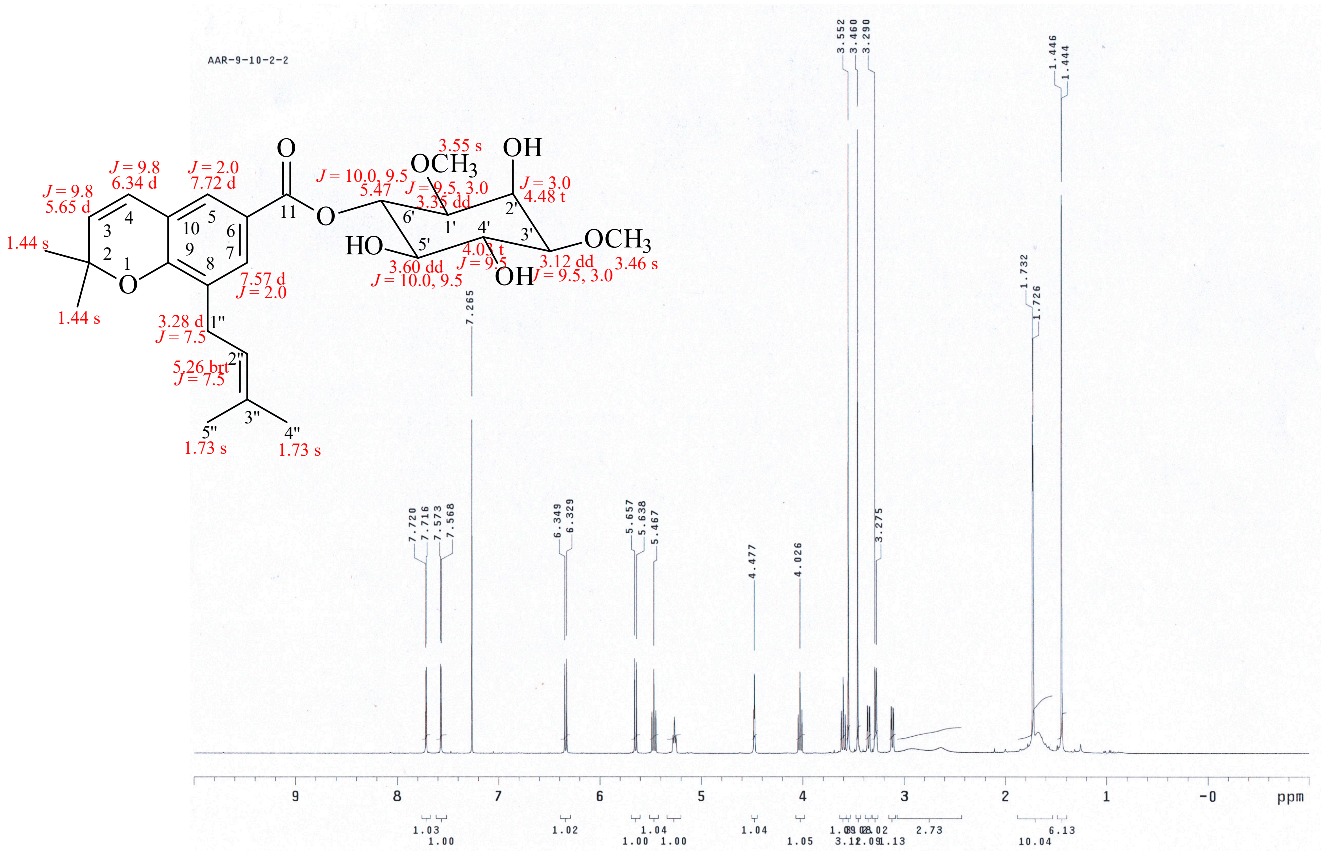

Figure S32. ^{1}H-NMR spectrum of Anodendrosin H (**4**).

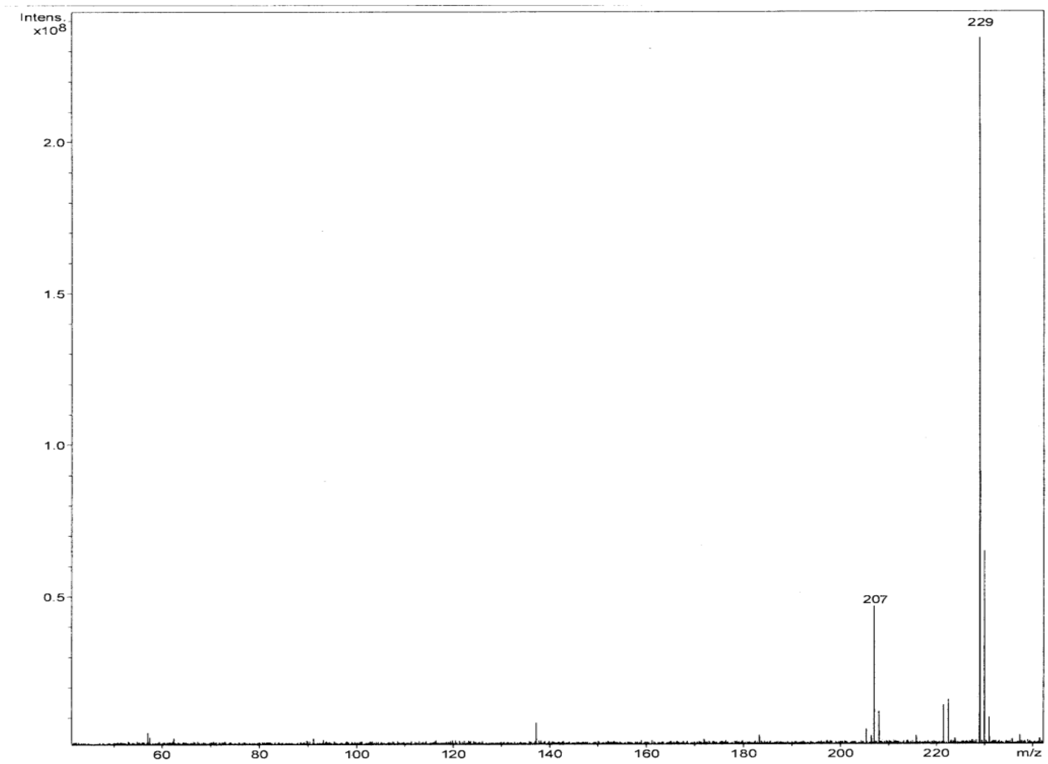

Figure S33. ESI-MS spectrum of 4-Hydroxy-3-prenylbenzoic acid (**5**).

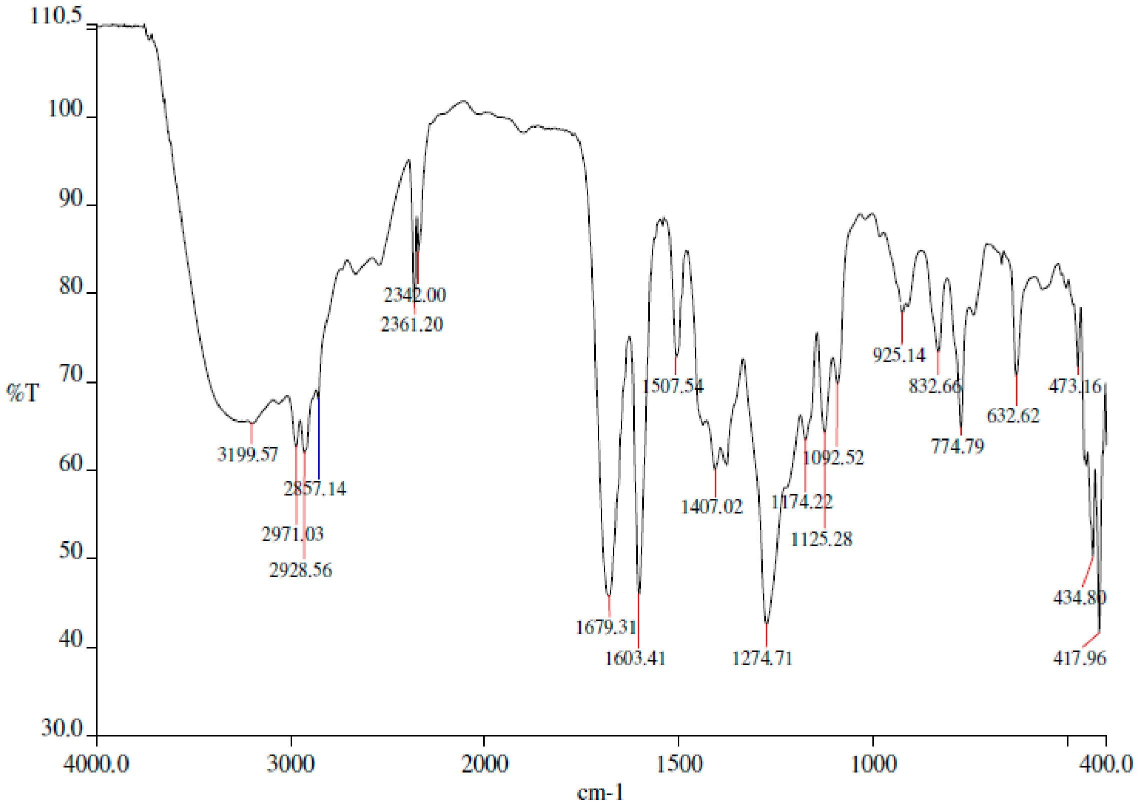

Figure S34. IR spectrum of 4-Hydroxy-3-prenylbenzoic acid (**5**).

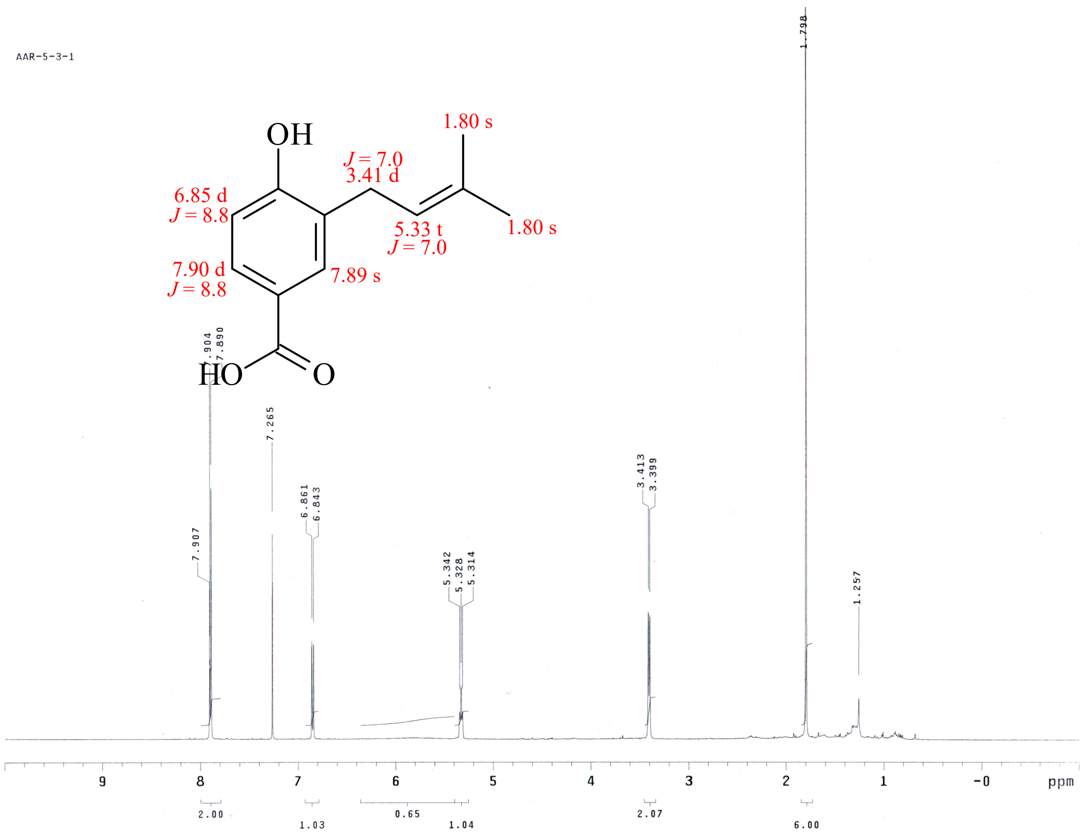

Figure S35. ^{1}H-NMR spectrum of 4-Hydroxy-3-prenylbenzoic acid (**5**).

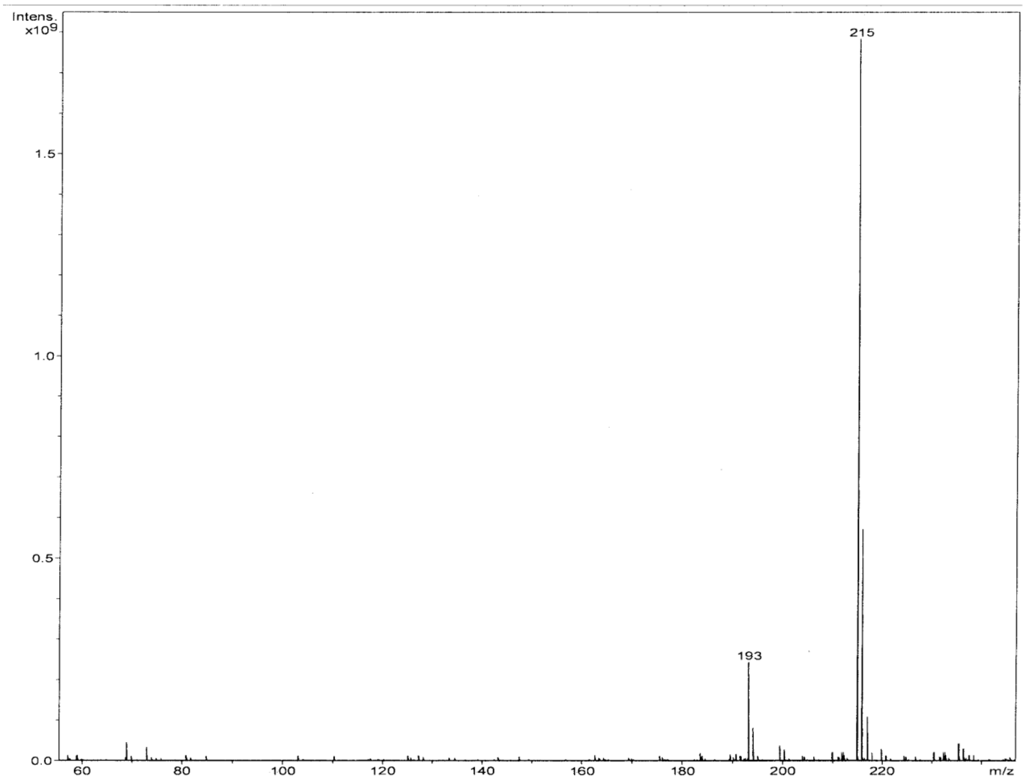

Figure S36. ESI-MS spectrum of Gelseminic acid (**6**).

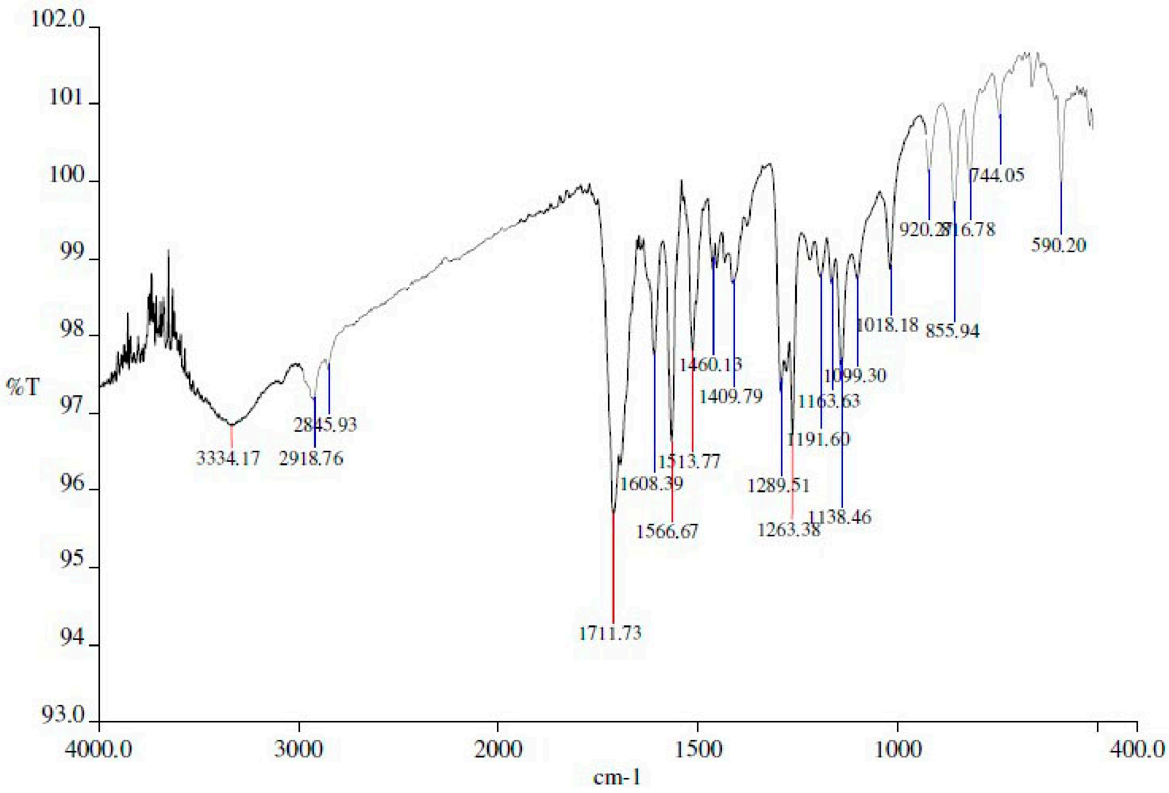

Figure S37. IR spectrum of Gelseminic acid (**6**).

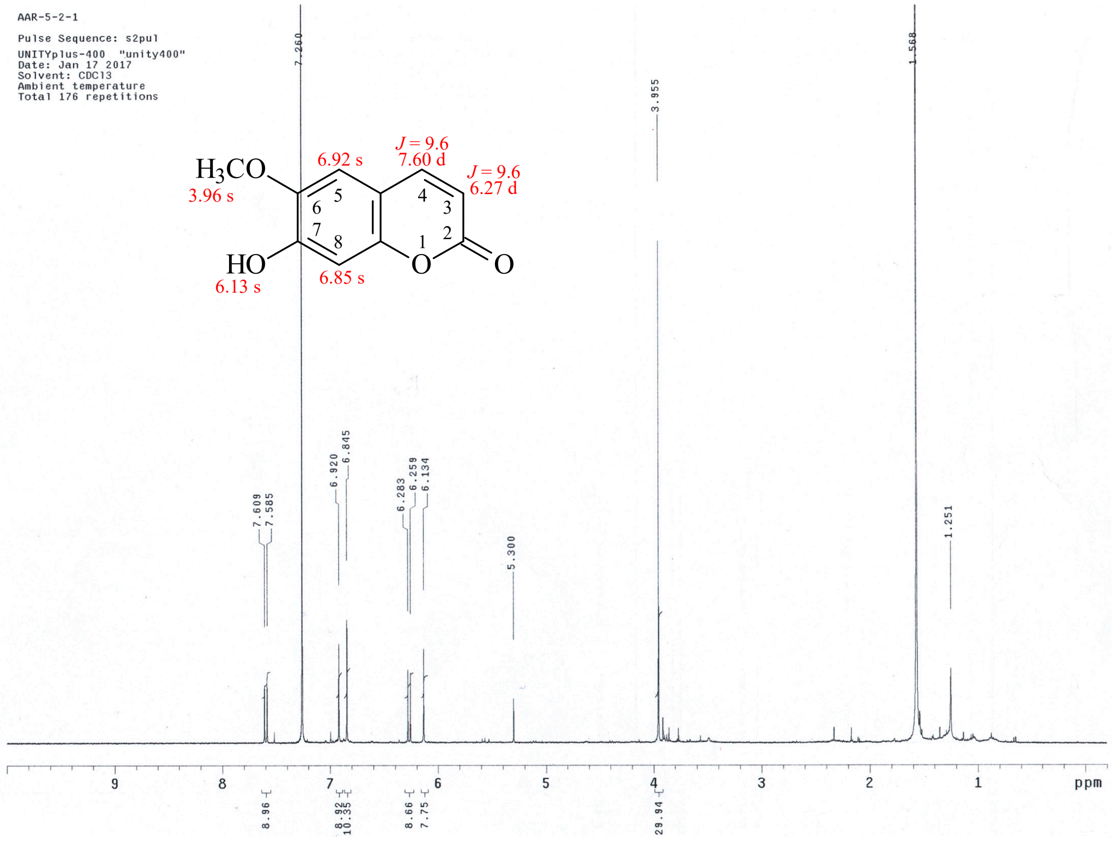

Figure S38. ^{1}H-NMR spectrum of Gelseminic acid (**6**).
